# Designing a zero-order energy transition model: How to create a new Starter Data Kit

**DOI:** 10.1016/j.mex.2023.102120

**Published:** 2023-03-12

**Authors:** Carla Cannone, Lucy Allington, Karla Cervantes Barron, Flora Charbonnier, Miriam Zachau Walker, Claire Halloran, Rudolf Yeganyan, Naomi Tan, Jonathan M Cullen, John Harrison, Long Seng To, Mark Howells

**Affiliations:** a Centre for Sustainable Transitions: Energy, Environment & Resilience (STEER), Loughborough University, United Kingdom; b Centre for Environmental Policy, Imperial College London, United Kingdom; c Department of Engineering, University of Cambridge, United Kingdom; d Department of Engineering Science, University of Oxford, United Kingdom

**Keywords:** Energy system modelling, Data collection tool, OSeMOSYS, clicSAND, U4RIA, open-access model, open-source, Climate Compatible Growth, data pipeline, Data2Deal, workflow, Data Collection and Manipulation Method for Starter Data Kits models

## Abstract

The Paris Agreement was signed by 192 Parties, who committed to reducing emissions. Reaching such commitments by developing national decarbonisation strategies requires significant analyses and investment. Analyses for such strategies are often delayed due to a lack of accurate and up-to-date data for creating energy transition models. The Starter Data Kits address this issue by providing open-source, zero-level country datasets to accelerate the energy planning process. There is a strong demand for replicating the process of creating Starter Data Kits because they are currently only available for 69 countries in Africa, Asia, and South America. Using an African country as an example, this paper presents the methodology to create a Starter Data Kit made of tool-agnostic data repositories and OSeMOSYS-specific data files. The paper illustrates the steps involved, provides additional information for conducting similar work in Asia and South America, and highlights the limitations of the current version of the Starter Data Kits. Future development is proposed to expand the datasets, including new and more accurate data and new energy sectors. Therefore, this document provides instructions on the steps and materials required to develop a Starter Data Kit.

• The methodology presented here is intended to encourage practitioners to apply it to new countries and expand the current Starter Data Kits library.
• It is a novel process that creates data pipelines that feed into a single Data Collection and Manipulation Tool (DaCoMaTool).
• It allows for tool-agnostic data creation in a consistent format ready for a modelling analysis using one of the available tools.

## Graphical abstract

**Figure:**
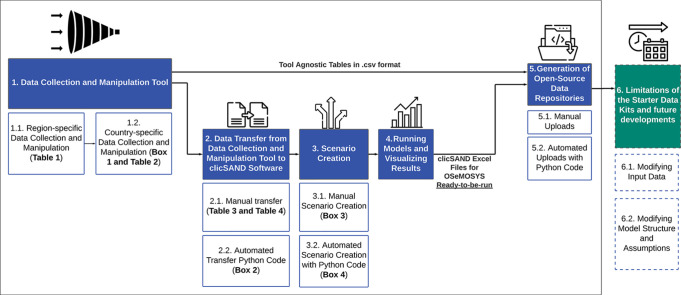

Specifications table

**Table:** 

Subject Area	Energy
More specific subject area	Energy System Modelling
Method name	Data Collection and Manipulation Method for Starter Data Kits models
Name and reference of original method	Not applicable
Resource availability	Annex A - Links to Zenodo Repositories Annex B - Methodology for Asian and South American Regions Annex C - Main Boxes, Tables and Useful Files

## Value of the Methodology

Decarbonisation strategies have been committed by 192 countries as part of the Paris Agreement with the aim of reducing global warming associated with greenhouse gas emissions [1]. Approximately 72% of those emissions are attributed to energy. The energy system is complex: it has long-term infrastructure, is subject to physical constraints and can be expensive. Data-driven, thermodynamically consistent, and up-to-date modelled scenarios can be used to estimate emissions and costs. However, the data needed for modelling may be time-consuming to collect, outdated, inconsistent, unreliable, and may come from proprietary sources. Additionally, certain software used to model energy systems may require specialist skills. Energy-system data compiled in the correct form for a given year can also be onerous to recompile a year later if the data sources have changed, or if the methods have not been documented. The Starter Data Kits overcome many of these issues by providing a set of open-source data and creating a repeatable method that uses available online data to produce ready-to-use outputs, such as an initial investment model. These outputs can be further treated and entered into an open-source model with an easy-to-use interface and available documentation, thereby giving users the option to rapidly adopt, adapt, update, and apply a model without needing a particular skill set. Such investment models can then be used for clear policy targets and loan applications, fast policy analysis, and slower burn academic processes.

The key scientific value of this paper is therefore the creation of a replicable and open-source method to collect and employ energy system data that can be used for credible energy modelling. This ensures the sustainability of reliable and accurate data-based modelling for well-informed policymaking, which may be useful for practitioners such as academics, civil servants, analysts, and policymakers.

The methodology presented for creating a Starter Data Kit and tool-agnostic data repositories has been tailored to Africa, Asia, and South America [2]. The added value of the data pipeline is that it can be easily adapted for any other country and aggregated to any region, therefore, anyone using this blueprint can create new energy system scenarios or even a new Starter Data Kit for a country of their choice.

The novel methodology for Starter Data Kits has been adapted for use with clicSAND software [3], the newest interface for OSeMOSYS - the Open-Source energy Modelling System [4]. However, it is essential to note that one of the main outputs of this methodology is an open-source collection of tool-agnostic data – a detailed description of the output repositories is provided in Section 5. Users can feed the country datasets as inputs to tools different to the ones presented in this paper, thereby improving the accuracy of the data by pointing to new sources or adding new data types. To date, the methodology has been used in connection with several other tools and studies, for example:

• To analyse and compare the electricity production, capacity, costs, and carbon emissions of six potential energy scenarios in Vietnam, for the United Kingdom Foreign, Commonwealth and Development Office and British Embassy in Hanoi [5]. Furthermore, using the Starter Data Kits in the modelling study allowed further exploration of the energy system, mainly power system flexibility. In a separate piece of work, additional data from the Starter Data Kits and the scenario results from [5] were modelled on a separate open-source tool to identify the potential baseload, peak load, curtailment and loss of load of each scenario previously studied [6];
• By academics at the University of Mauritius to develop new Starter Data Kits for their country. Their aim is to promote regional collaboration between the African Small Island Developing States (SIDS) for collective effort to encourage decarbonisation and sustainable development, and investigate explorative energy scenarios [7,8,9,10];
• In combination with other tools such as the European Climate Calculator model (EUCalc) for a country model of Qatar [11];
• In China, the Starter Data Kits will be combined with the China 2050 Calculator (jointly developed by China and the UK) to deduce China's energy consumption in 2050, providing a basis for Chinese policymakers to formulate environmental policies and help China achieve its carbon neutrality goals.
• In Kenya, the Starter Data Kits have provided the foundation Reference Energy System (RES) for two energy planning tools for the Kenyan government: a standalone power system model indicating new investment requirements in powerbase generation capacity and an integrated whole energy system model. Applying this methodology resulted in saved computational time but ensured the critical dynamics of supply and demand variability were maintained.
• Together with an investigation of the potential for accelerated Electric Vehicle deployment in Kyrgyzstan [12]; and
• To build a starting point model for Armenia and analyse alternative development strategies.

## Paper structure

**Fig. 1:**
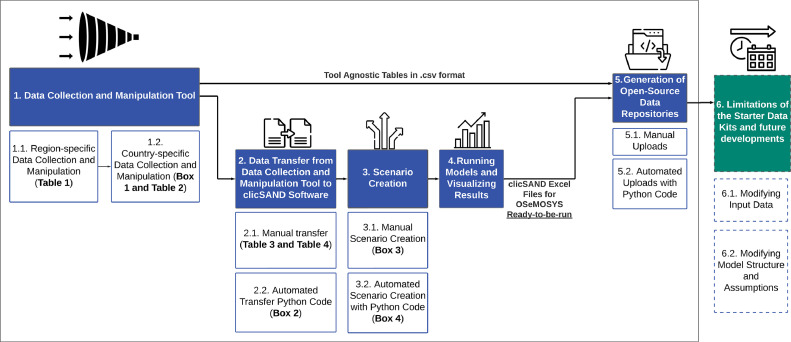
Description of the complete process to develop a Starter Data Kit for any country – an open-source data repository made of tool-agnostic data and OSeMOSYS-specific data files ready-to-be-run with clicSAND software. **Boxes** and **Tables,** which include step-by-step instructions to replicate the methodology performed, can be found in Annex C. .

The remainder of the paper is structured as presented in Fig. 1. Section 1 describes the main activities to be carried out with the Data Collection and Manipulation Tool (DaCoMaTool). This first section contains two separate subsections for the region- and country-specific data. Once all data have been collected and put into a readable format, they are transferred from the DaCoMaTool to the clicSAND software. Section 2 describes two alternative ways of performing this task: a manual and an automated process. Three copies of the output, the Base SAND file, are created, in a manual or automated way, to develop three scenarios for each country: Fossil Future, Least Cost, and Net Zero (described in Section 3). Section 4 then explains how to run a model using the clicSAND software and how to visualise the results. Finally, Section 5 describes how to create open-source data repositories which include both tool-agnostic tables in .csv format and OSeMOSYS-specific data files for the three scenarios modelled. Section 6 is an addition to this methodology which describes future work and potential improvements to the Starter Data Kits collection. Such work is expected to include the expansion of the data kits to include transport and heating data.

## Data collection and manipulation tool (DaCoMaTool)

The DaCoMaTool is an original tool designed to accelerate and automate the Starter Data Kit development process and the creation of consistent tool-agnostic data repositories. The DaCoMaTool should be seen as a funnel with a filter, where data coming from multiple sources (therefore most probably inconsistent in its format) are collected and manipulated in a single platform to produce consistent and ready-to-use open-source data. It is an Excel workbook made of multiple tabs, where the user can collect and manipulate data from various sources and organise it into an easy-to-use format in a single open collection. As shown in Fig. 1, this output data, made available as part of the open-source data repositories (Step 5), is ready for use by analysts and is energy-modelling-software agnostic. As explained in the previous chapter, the user is not required to use OSeMOSYS and can instead download these datasets for use with other tools and different analyses.

Nevertheless, as an additional value, DaCoMaTool was also designed to be fully compatible with clicSAND software [3] for the Open-Source energy Modelling SYStem – OSeMOSYS [4] to be able to compile a Starter Data Kit from scracth for any country in the world. This paper presents, as an example, the steps to create a Starter Data Kit for a country in Africa, starting with the parameters for the Excel workbook. Further information on the definition, and the meaning of each of the following parameters described below, is freely available on the OSeMOSYS course hosted on the Open Learn Create (OLC) platform for Windows and macOS users [13,14].

### Region-specific data collection and manipulation

The method and source data recorded in the Data Collection and Manipulation File (which can be found in the Zenodo repository with the links provided in Annex C) for a country contains data that will remain the same for all countries in that region. Therefore, by way of example, Africa's method is the same for all its continental countries. Below is a summary of where the region-specific data for Africa were sourced and how they were manipulated. Annex B includes this information for the Asian and South American regions. Further details on the data sources can be found in the Data in Brief article [2] and in the country Data Collection and Manipulation file, under the column “Comments” in each tab.

#### Depreciation method, discount rate and year split

The Discount Rate and the Depreciation Method were set to 0.1 and 1 respectively, as conventional default values. The user has the freedom to change it according to their needs using the clicSAND Interface or similar tools. In this model, the year was initially divided into four timeslices, representing two periods of six months (two representative seasons) each of which has similar demand, further sub-divided into day and night periods, called: Summer Day (SD), Summer Night (SN), Winter Day (WD), Winter Night (WN). However, in clicSAND Interface it is possible to define up to 96 timeslices, so these initial data were manipulated to obtain a 24-hour representation of a reference day for each of SD, SN, WD, and WN (24 h each * 4 = 96 timeslices). Therefore, each year is divided into 96 periods instead of the previous 4. It was assumed each season has an equal length, with an average hourly split per season (24 h representative). Therefore obtaining: 4 Seasons/year * 24 h of a representative day/season = 96 Timeslices/Year. Each Timeslice represents an equal fraction of the Year in the following way, defined as the Year Split: 1 Year / 96 Timeslices = 0.0104. More information on the definition of timeslices can be found in “Hands-on 2” [15] of the online course on “Energy and Flexibility Modelling” hosted on the OLC platform of the Open University [14]. However, the user can adjust the number of timeslices depending on the specific needs, by following the instructions presented in these tutorials [16,17].

#### Technologies costs – Africa

The Electricity Model Base for Africa (TEMBA) report was used to collect the costs of refinery and transmission and distribution technologies [18,19]. Costs for renewables were taken from IRENA (2021) Planning & Prospects for Renewable Power: Eastern & Southern Africa [20] – this provides 5-yearly projected costs to 2040, with linear change assumed between datapoints and costs assumed constant after 2040. The costs of offshore wind were taken from IRENA (2019) The Future of Wind [21]. Costs for fossil power plants were taken from IRENA (2018) Planning & Prospects for Renewable Power: West Africa [22] (because the gas and coal costs were deemed less realistic in the 2021 report). Generic costs of transport and heating technologies were taken from Terpilowski Gill (2020) Decarbonising the Laotian Energy System [23]. The costs of energy efficiency technologies were estimated based on the costs of coal power plants – see [24]. Costs of stoves were taken from Okolo O, Teng H. (2017) Analysing Nigeria's Energy system in light of the UN's Sustainable Development Goals [25]. Costs of renewables with storage were estimated by combining the standard cost from the IRENA report with an estimated storage cost based on the National Renewable Energyb Laboratory (NREL) 2020 Annual Technology Baseline [26] – see calculations in this file [27]. The oil price was taken directly from that U.S. EIA. (2020) Assumptions to the Annual Energy Outlook 2020: International Energy Module [28], extended to 2070 by extrapolating the rate of change from 2045 to 2050 for the years 2050–2070 – see calculations in this file [29].That oil price is taken to be the domestic oil price. This domestic oil price was then increased by 10% to reflect the cost of importation (as done for TEMBA [18]), to give the imported oil price. This imported oil price is then multiplied by 0.8 to give the price of imported Heavy Fuel Oil (HFO) (so the imported HFO price is 80% of the imported oil price), and by 1.333 to give the price of imported Light Fuel Oil (LFO) (so the imported LFO price is 133% of the imported oil price) - again as done for TEMBA [18]. Prices of other fuels were taken from [22].

#### Input & output activity ratios

Region-specific efficiencies for power transmission and distribution were used. For Africa, efficiencies of power plants were taken from IRENA (2018) Planning & Prospects for Renewable Power: West Africa [22], and efficiencies of stoves were taken from Okolo O, Teng H. [25].

#### Capacity to activity unit, operational life & capacity factors

Default OSeMOSYS values were used for the Capacity-to-Activity unit; for more information on default data see [30]. Operational lifetimes for the facilities were taken from the same reports used for technology costs in each region. Capacity factors for fossil, biomass and nuclear power plants were also taken from those reports. Country-specific capacity factors for wind, solar, and hydropower technologies were used, sourced from the PLEXOS dataset [31] (based on Renewables Ninja [32]) and global NREL datasets [33] where necessary (detailed in the following sections).

#### Emissions factors

Emissions factors were sourced from Table 1.3 of the IPCC report [34].

### Country-specific data collection and manipulation

Box 1, available in Annex C, presents an example of creating a Starter Data Kit for an African country. The box is organized into a series of instructions, or a “How-to guide” to allow for the method to be applied by users to other country analyses.

## Data transfer from data collection and manipulation tool to clicSAND software

After all the data have been collected in DaCoMaTool (based on the instructions of Box 1 of Annex C), the next step is to transfer all the data from the DaCoMaTool to the modelling tool selected for the analysis. Here, the example of transferring this dataset to clicSAND software is presented, which is a user-friendly interface for OSeMOSYS users. More information on the architecture, the specific functionalities and the installation process for this software can be found in Cannone et al. [3]. A step-by-step course is freely available on the OLC platform of the Open University [14]. This course guides the user through the steps required to build a simple OSeMOSYS model using clicSAND software [13]. At the end of the course, the user receives a certificate of completion. clicSAND software is available also for macOS users (here [35] and [36]).

In clicSAND software, the user adds the data in an Excel workbook – called SAND Interface – which contains all the OSeMOSYS parameters needed to create a model that the solver can process to find the optimal solution [37]. The following instructions describe how to transfer data into clicSAND software. Option 2.1 describes how to do this manually using copy and paste without any programming language; Option 2.2 describes how to do this more efficiently using Python. The authors suggest Option 2.2 only if multiple Starter Data Kits are being created simultaneously: for example, if the user creates a Starter Data Kit for each country of a selected region. Using the Python code will accelerate the transfer and reduce the risk of human error while copy-pasting. On the other hand, if the user is working on a single country Starter Data Kit and has no previous knowledge of Python, Option 2.1 should be used.

### Manual transfer

The first data that need to be added are the Regional Specific Data. The user has two options:

a) Download a blank Excel SAND Interface from this online repository [38] and build the model from scratch, involving defining new technologies and sets, linking them as per OSeMOSYS convention, and adding the data.
b) Download one of the pre-filled SAND files freely available on Zenodo for Africa [39], Asia [40], and South America [41]. These files already have all the Starter Data Kit technologies defined, and all the regional data added.

The authors strongly advise choosing option (b) even if the user is building a Starter Data Kit for a country not in Africa, Asia, or South America. Using one of the pre-filled files will dramatically speed up the process as the entire model is already built. The user will need to update the region- and country-specific data rather than add all the scratch information.

However, if the user is interested in creating a model that is not meant to be a Starter Data Kit, option (a) would be more appropriate. For instructions on how to compile an OSeMOSYS model from scratch using SAND Interface (as part of the clicSAND software), please refer to the freely available courses available for Windows and Mac users, [13] and [14] respectively.

Instead, if the user wants to create a Starter Data Kit using option (b), the following steps should be performed to transfer the data, as summarized in Table 3 and Table 4 (available in Annex C). It is important to note that data should be copy-pasted from the Data Collection and the Manipulation Tool to the SAND Excel Interface using the “paste values” function. The authors suggest renaming the downloaded SAND Excel file as “New_your_country_name_Base_SAND” and then adding the regional (Table 3) and country-specific data (Table 4). The description of each code used in the Tables below and the OSeMOSYS parameters can be found in the first tab called “Naming” in the SAND Interface Excel file. However, the authors strongly encourage users who want to create a Starter Data Kit to complete the online course to acquire the basic skills needed.

### Automated transfer with python code

In Box 2 in Annex C the step-by-step instructions are presented to transfer the data from the DaCoMaTool to clicSAND software in an automated way using a Python code.

## Scenario creation

At the end of Section 2, the user will have completed transferring the data from the Data Collection and Manipulation file to their “New_your_country_name_Base_SAND”, which will include all the regional and country-specific data. On this file, no additional constraints are applied; therefore, if the user runs this model, the OSeMOSYS code will identify the optimal solution of the problem. This section will explain the steps needed to recreate the three scenarios available for each Starter Data Kit: Fossil Future, Least Cost, and Net Zero Scenario. For more information on the definition and the idea behind each of these scenarios, the reader should refer to the Data in Brief publication on the Starter Data Kits [2].

To speed up the process of applying constraints on the Base SAND file, a support Excel template called “Scenarios Constraints” [42] was used, freely available for download on each Starter Data Kit Zenodo repository. Instructions are available in the workbook. This workbook has four tabs:

• Input Data – add initial data, such as Specified Annual Demands and the grid and off-grid extension split.
• Fossil Future – add constraints using TotalAnnualMaxCapacityInvestment and TotalTechnologyAnnualActivityUpperLimit parameters.
• Least Cost – add constraints and run the model two times to constraint wind and solar
• Net Zero – add a constraint to nuclear, primary fossil fuel and biomass production technologies. The model needs to be run two times.

Hereafter, the instructions on applying constraints to the Base SAND file will be shared to create the three scenarios. However, an intermediate run should be performed to complete a Least Cost and Net Zero Scenarios. Box 3 presents the manual way of performing this task while Box 4 the automated alternative using Python codes. The user should consult Section 4 for more instructions on running and visualising results using clicSAND software for OSeMOSYS.

## Running models and visualising results

clicSAND software was used to run the three scenarios and SAND Excel data files. clicSAND software includes two free powerful software, namely GLPK and CBC. The following resources can be used to learn how to run a model and visualise results using this software:

• The most updated OLC free online course version is called “Energy and Flexibility Modelling” OLC for Windows and Mac Users [13,43].
• SoftwareX paper on clicSAND software functionalities, architecture, and documentation material [35].
• Video tutorial on how to install and use the latest version of the software called
• clicSAND 3.0 [44,45,46], as well as, how to run the model using the OSeMOSYS Cloud online platform [47,48]. This option drastically reduces the computational time and the RAM requirements of the operating machine.

## Generation of open-source data repositories

### Manual uploads

A Zenodo repository has been created for each country, containing the following documents, where **(TA)** is tool-agnostic and **(OS)** is OSeMOSYS-specific. The full list of Zenodo repositories for each of the existing Starter Data Kits can be found on the Climate Compatible Growth website [44] and is also available in Annex A.

• Table1_<COUNTRY>.csv: Installed Power Plants Capacity in The Country **(TA)**.
• Table2_<COUNTRY>.csv: Techno-economic parameters of power generation technologies **(TA)**.
• Table3_<COUNTRY>.csv: Projected costs of renewable power generation technologies for selected years to 2050 **(TA)**.
• Table4_<COUNTRY>.csv: Techno-economic parameters for transmission and distribution technologies **(TA)**.
• Table5_<COUNTRY>.csv: Techno-economic parameters for refinery technologies **(TA)**.
• Table6_<COUNTRY>.csv: Fuel price projections to 2050 **(TA)**.
• Table7_<COUNTRY>.csv: Fuel-specific CO2 Emission Factors **(TA)**.
• Table8_<COUNTRY>.csv: Estimated Renewable Energy Potentials **(TA)**.
• Table9_<COUNTRY>.csv: Estimated Fossil Fuel Reserves **(TA)**.
• A `references. bib` file, which contains all the data sources **(TA)**.
• A SAND file: “<COUNTRY> Base SAND.xlsm” **(OS)**.
• A SAND file: “<COUNTRY> FF SAND.xlsm” **(OS)**.
• A SAND file: “<COUNTRY> LCv2 SAND.xlsm” **(OS)**.
• A SAND file: “<COUNTRY> NZv2 SAND.xlsm” – if this scenario was feasible for the selected country **(OS)**.
• Data Collection and Manipulation File: “New_Country_Name_Data Collection.xlsx” **(TA)**.
• Template to create scenarios: “Country_Name_Scenario_Constraints .xlsx” File **(OS)**.

It is important to note that Tables 1–9 in each repository are tool and interface agnostic, meaning that these data can be used with other tools or interfaces The user is therefore not locked into using OSeMOSYS or clicSAND software. Nevertheless, the SAND files have been added to the repository to speed up the uptake of the Starter Data Kits already created.

To extend an existing Starter Data Kit, it is recommended to download the SAND files available in the Country repository and start building on these. For example, suppose the renewable constraints that have been applied are not consistent with the local reality in the country or the most updated government plans. In that case, the Scenario Constraints support file can be used to adjust that constraint quickly; and this can then be added to a new version of the Scenario file, rerun, and the results compared.

This is just one of many adjustments that can be made to the Starter Data Kits. These Starter Data Kits are zero-order, therefore starting point, energy models that accelerate the energy modelling process by avoiding time-consuming activities such as model creation, design, data collection, and scenario development. In the next section, some suggestions for extending the Starter Data Kits will be presented and future developments for new kits.

### Automated uploads with python code

There is an option for users who have created model results for several countries and want to share them on Zenodo via batch upload. This requires running a script to craft the batch upload. The scripts can be found in a Github repository [49], which contains its own instructions for use and can be forked (i.e., copied to a Github user's repository for customization) in case adjustments to the files included or file paths are needed.

## Limitations of the starter data kits and future developments

Potential extensions to the Starter Data Kits to improve their accuracy and extend their data coverage are detailed below. Works using the Starter Data Kits should always cite the Data in Brief paper, the MethodsX, and Zenodo repository for the selected country. Those wishing to conduct their analyses using these data as a starting point should consult the country-specific SAND files available on Zenodo for the most accurate and final input assumptions for each country. Opportunities to build on the Starter Data Kit model through extensions could include modifying input data, the model structure, and the main assumptions. The authors suggest that new data types could be added to the methodology in future iterations of the Starter Data Kits.

### Modifying the input data

Currently, several model assumptions use generic values. Thus, there is potential for such assumptions to be improved by sector, country, or region. Details of such improvements are the following:

• The same demand profile is used across all sectors, so this could be adjusted for each sector to account for peaks occurring at different times in different energy sectors.
• Generic costs are used for cooking, transport, and heating technologies, so finding more country- or region-specific costs could add value.
• Only CO_2_ emissions were considered, so adding in emissions factors for methane, or other GHGs would be an improvement.
• Residual capacity is based on a global power model (PLEXOS [50]). These sources have gaps that could be reduced by comparing the data with government data or detailed country sources. Planned projects should also be added.

### Modifying model structure and assumptions

The model scope is growing regarding countries, regions and supporting technologies (e.g., storage). Thus, the assumptions in the model and the structure of the data and calculations need to include this growing complexity. Some areas where change would be beneficial, and their proposed changes are the following:

• Split demands within sectors further and potentially add others such as agriculture.
• Demands are treated in an introductory manner – it would be valuable to improve these by converting the transport demand to consider final transport demand in vehicle/km unit. A more comprehensive range of transport technologies could be added, and country-specific data on modal share could be sourced (currently, an assumption is made for each country based on regional data).
• There is a uniform approach to time-slicing with four seasons, each split into day and night, resulting in 96 timeslices, as further explained in Section1.1.1. Increasing the accuracy of time representation to consider the seasonality in different countries would be an improvement. This would require adjusting the capacity factor and specified demand profiles, and potentially the year split depending on the approach.
• Currently, only one region is used, so perhaps for countries with significant geographic variability, a multi-region approach could improve accuracy.
• Storage and flexibility are considered in a simple manner. Thus, specific storage technologies could be added to the model.
• Other technologies could be added, e.g., interconnector projects (existing and planned) or LNG terminals.
• There are gradual investment constraints on demand-side technologies and renewable energy potentials in the scenarios. However, in-country knowledge could limit yearly investment in power plant technologies and make capacity expansion more realistic. It would be equally essential to improve the consideration of the time taken to approve and construct each power plant technology, most likely using total annual maximum capacity investment in OSeMOSYS.
• Imports and exports are modelled in a simple manner. It is assumed that the country can continue importing and exporting power at the same levels as in 2020, with no costs considered. To improve this, the option could be provided to scale imports and exports with demand and to add an electricity price that would allow the model to import and export as is economical. Interconnectors are also not considered.
• It would be valuable to improve the country's policies and plans. For example, the model currently allows investment in any technically feasible technologies in the country (although nuclear is turned off in all scenarios). However, it may be that certain technologies are more or less likely to feature in the country due to policy goals conflicts with land and water use.

## Funding

This paper has been written with the support of the Climate Compatible Growth Programme (#CCG) of the UK's Foreign, Commonwealth & Development Office (FCDO). The views expressed in this paper do not necessarily reflect the UK government's official policies.

## CRediT authorship contribution statement

**Carla Cannone:** Conceptualization, Data curation, Investigation, Methodology, Formal analysis, Validation, Visualization, Writing – original draft. **Lucy Allington:** Conceptualization, Data curation, Investigation, Visualization, Validation, Writing – original draft. **Karla Cervantes Barron:** Data curation, Validation, Visualization, Writing – original draft. **Flora Charbonnier:** Data curation, Validation, Writing – original draft. **Miriam Zachau Walker:** Data curation, Validation, Writing – original draft. **Claire Halloran:** Data curation, Validation, Writing – original draft. **Rudolf Yeganyan:** Writing – review & editing. **Naomi Tan:** Writing – review & editing. **Jonathan M Cullen:** Writing – review & editing. **John Harrison:** Writing – review & editing. **Long Seng To:** Writing – review & editing. **Mark Howells:** Writing – review & editing.

## Declaration of Competing Interest

The authors declare that they have no known competing financial interests or personal relationships which have or could be perceived to have influenced the work reported in this article.

## Data Availability

Links to data repository are available in the paper and in the Supplementary files.

## Appendix Supplementary materials

Image, application 1

Image, application 2

Image, application 3
