# Supplementary material for: Designing a zero-order energy transition model: How to create a new Starter Data Kit
Source: MethodsX. 2023 Mar 12;10:102120. doi: 10.1016/j.mex.2023.102120 (PMC10050781; doi:10.1016/j.mex.2023.102120)
Supplement: Supplementary file 1 [file mmc1.docx]

**Designing a zero-order energy transition model: a guide for creating a Starter Data Kit**

| **Title** | Designing a zero-order energy transition model: how to create a new Starter Data Kit |
| --- | --- |
| **Authors** | Carla Cannone* [1,2], Lucy Allington [1], Karla Cervantes Barron [3], Flora Charbonnier [4], Miriam Zachau Walker [4], Claire Halloran [4], Rudolf Yeganyan [1,2], Naomi Tan [1,2], Jonathan M Cullen [3], John Harrison [1], Long Seng To [1] and Mark Howells [1,2]. |
| **Affiliations** | 1. Centre for Sustainable Transitions: Energy, Environment & Resilience (STEER), Loughborough University, United Kingdom  2. Imperial College London, United Kingdom  3. University of Cambridge, United Kingdom  4. University of Oxford, United Kingdom |
| **Corresponding Author's email address** | C.Cannone@lboro.ac.uk |
| **Keywords** | Energy System Modelling  Data Collection Tool  OSeMOSYS  clicSAND  U4RIA |
| **Direct Submission or Co-Submission**  *Co-submissions are papers that have been submitted alongside an original research paper accepted for publication by another Elsevier journal* | *Co-Submission* |

**ABSTRACT**

The Paris Agreement was signed by 192 Parties, who committed to reducing emissions. Reaching such commitments by developing national decarbonisation strategies requires significant analyses and investment. Analyses for such strategies are often delayed due to a lack of accurate and up-to-date data for creating energy transition models. The Starter Data Kits address this issue by providing open-source, zero-level country datasets to accelerate the energy planning process. There is a strong demand for replicating the process of creating Starter Data Kits because they are currently only available for 69 countries in Africa, Asia, and South America. Using an African country as an example, this paper presents the methodology to create a Starter Data Kit from data collection to the creation of tool-agnostic data repositories. The paper illustrates the steps involved, provides additional information for conducting similar work in Asia and South America, and highlights the limitations of the current version of the Starter Data Kits. Future development is proposed to expand the datasets, including new and more accurate data and new energy sectors.

**SPECIFICATIONS TABLE**

| **Subject Area** | Energy |
| --- | --- |
| **More specific subject area** | Energy System Modelling |
| **Method name** | Data Collection and Manipulation Method for Starter Data Kits models |
| **Name and reference of original method** | Not applicable |
| **Resource availability** | Annex A - Links to Zenodo Repositories  Annex B - Methodology for Asian and South American Regions  Annex C - Main Boxes, Tables and Useful Files |

**Annex A – Full list of Zenodo repositories and preprint papers per country**

| **Region** | **Country** | **Zenodo Dataset** | **ResearchSquare Pre-Print** |
| --- | --- | --- | --- |
| Africa | Algeria | <https://doi.org/10.5281/zenodo.4728143> | <https://www.researchsquare.com/article/rs-478421/v2> |
| Africa | Angola | <https://doi.org/10.5281/zenodo.4650810> | <https://www.researchsquare.com/article/rs-478581/v2> |
| Africa | Benin | <https://doi.org/10.5281/zenodo.4725486> | <https://www.researchsquare.com/article/rs-478594/v2> |
| Africa | Botswana | <https://doi.org/10.5281/zenodo.4650986> | <https://www.researchsquare.com/article/rs-478620/v2> |
| Africa | Burkina Faso | <https://doi.org/10.5281/zenodo.4650942> | <https://www.researchsquare.com/article/rs-478764/v2> |
| Africa | Burundi | <https://doi.org/10.5281/zenodo.4725445> | <https://www.researchsquare.com/article/rs-478806/v2> |
| Africa | Cameroon | <https://doi.org/10.5281/zenodo.4650822> | <https://www.researchsquare.com/article/rs-478850/v2> |
| Africa | Central African Republic | <https://doi.org/10.5281/zenodo.4650968> | <https://www.researchsquare.com/article/rs-478906/v2> |
| Africa | Chad | <https://doi.org/10.5281/zenodo.4725466> | <https://www.researchsquare.com/article/rs-478927/v2> |
| Africa | Côte d'Ivoire | <https://doi.org/10.5281/zenodo.4737634> | <https://www.researchsquare.com/article/rs-493226/v1> |
| Africa | Democratic Republic of the Congo | <https://doi.org/10.5281/zenodo.4737640> | <https://www.researchsquare.com/article/rs-493235/v1> |
| Africa | Djibouti | <https://doi.org/10.5281/zenodo.4725462> | <https://www.researchsquare.com/article/rs-479210/v2> |
| Africa | Egypt | <https://doi.org/10.5281/zenodo.4652804> | <https://www.researchsquare.com/article/rs-479263/v2> |
| Africa | Equatorial Guinea | <https://doi.org/10.5281/zenodo.4650904> | <https://www.researchsquare.com/article/rs-479310/v2> |
| Africa | Eritrea | <https://doi.org/10.5281/zenodo.4725456> | <https://www.researchsquare.com/article/rs-479568/v2> |
| Africa | Eswatini (fmr. "Swaziland") | <https://doi.org/10.5281/zenodo.4737638> | <https://www.researchsquare.com/article/rs-493243/v1> |
| Africa | Ethiopia | <https://doi.org/10.5281/zenodo.4650876> | <https://www.researchsquare.com/article/rs-479603/v2> |
| Africa | Gabon | <https://doi.org/10.5281/zenodo.4737642> | <https://www.researchsquare.com/article/rs-493249/v1> |
| Africa | Gambia | <https://doi.org/10.5281/zenodo.4651140> | <https://www.researchsquare.com/article/rs-479641/v2> |
| Africa | Ghana | <https://doi.org/10.5281/zenodo.4725480> | <https://www.researchsquare.com/article/rs-479778/v2> |
| Africa | Guinea | <https://doi.org/10.5281/zenodo.4725454> | <https://www.researchsquare.com/article/rs-480013/v3> |
| Africa | Guinea-Bissau | <https://doi.org/10.5281/zenodo.4650850> | <https://www.researchsquare.com/article/rs-480393/v1> |
| Africa | Kenya | <https://doi.org/10.5281/zenodo.4650874> | <https://www.researchsquare.com/article/rs-480458/v1> |
| Africa | Lesotho | <https://doi.org/10.5281/zenodo.4650866> | <https://www.researchsquare.com/article/rs-480748/v1> |
| Africa | Liberia | <https://doi.org/10.5281/zenodo.4650794> | <https://www.researchsquare.com/article/rs-480654/v1> |
| Africa | Libya | <https://doi.org/10.5281/zenodo.4650920> | <https://www.researchsquare.com/article/rs-481132/v1> |
| Africa | Malawi | <https://doi.org/10.5281/zenodo.4652798> | <https://www.researchsquare.com/article/rs-479507/v2> |
| Africa | Mali | <https://doi.org/10.5281/zenodo.4725447> | <https://www.researchsquare.com/article/rs-479627/v2> |
| Africa | Mauritania | <https://doi.org/10.5281/zenodo.4650914> | <https://www.researchsquare.com/article/rs-479591/v2> |
| Africa | Morocco | <https://doi.org/10.5281/zenodo.4725482> | <https://www.researchsquare.com/article/rs-480023/v2> |
| Africa | Mozambique | <https://doi.org/10.5281/zenodo.4650902> | <https://www.researchsquare.com/article/rs-481070/v1> |
| Africa | Namibia | <https://doi.org/10.5281/zenodo.4652808> | <https://www.researchsquare.com/article/rs-481002/v1> |
| Africa | Niger | <https://doi.org/10.5281/zenodo.4725476> | <https://www.researchsquare.com/article/rs-480051/v2> |
| Africa | Nigeria | <https://doi.org/10.5281/zenodo.4728145> | <https://www.researchsquare.com/article/rs-480085/v2> |
| Africa | Congo (Congo-Brazzaville) | <https://doi.org/10.5281/zenodo.4651133> | <https://www.researchsquare.com/article/rs-479154/v2> |
| Africa | Rwanda | <https://doi.org/10.5281/zenodo.4652800> | <https://www.researchsquare.com/article/rs-480847/v1> |
| Africa | Senegal | <https://doi.org/10.5281/zenodo.4725484> | <https://www.researchsquare.com/article/rs-480122/v2> |
| Africa | Sierra Leone | <https://doi.org/10.5281/zenodo.4725544> | <https://www.researchsquare.com/article/rs-480371/v2> |
| Africa | Somalia | <https://doi.org/10.5281/zenodo.4725474> | <https://www.researchsquare.com/article/rs-480695/v1> |
| Africa | South Africa | <https://doi.org/10.5281/zenodo.4652802> | <https://www.researchsquare.com/article/rs-480636/v1> |
| Africa | South Sudan | <https://doi.org/10.5281/zenodo.4725468> | <https://www.researchsquare.com/article/rs-479969/v2> |
| Africa | Sudan | <https://doi.org/10.5281/zenodo.4725460> | <https://www.researchsquare.com/article/rs-479952/v2> |
| Africa | Tanzania | <https://doi.org/10.5281/zenodo.4652806> | <https://www.researchsquare.com/article/rs-481182/v1> |
| Africa | Togo | <https://doi.org/10.5281/zenodo.4725451> | <https://www.researchsquare.com/article/rs-480160/v2> |
| Africa | Tunisia | <https://doi.org/10.5281/zenodo.4725458> | <https://www.researchsquare.com/article/rs-480566/v1> |
| Africa | Uganda | <https://doi.org/10.5281/zenodo.4652795> | <https://www.researchsquare.com/article/rs-480512/v1> |
| Africa | Zambia | <https://doi.org/10.5281/zenodo.4725470> | <https://www.researchsquare.com/article/rs-480042/v2> |
| Africa | Zimbabwe | <https://doi.org/10.5281/zenodo.4650816> | <https://www.researchsquare.com/article/rs-479655/v2> |
| Asia | Cambodia | <https://doi.org/10.5281/zenodo.5139538> | <https://www.researchsquare.com/article/rs-757472/v1> |
| Asia | Indonesia | <https://doi.org/10.5281/zenodo.4926858> | <https://www.researchsquare.com/article/rs-757493/v1> |
| Asia | Laos | <https://doi.org/10.5281/zenodo.4926880> | <https://www.researchsquare.com/article/rs-757542/v1> |
| Asia | Malaysia | <https://doi.org/10.5281/zenodo.5139480> | <https://www.researchsquare.com/article/rs-757581/v1> |
| Asia | Myanmar (formerly Burma) | <https://doi.org/10.5281/zenodo.5139484> | <https://www.researchsquare.com/article/rs-757622/v1> |
| Asia | Philippines | <https://doi.org/10.5281/zenodo.5139542> | <https://www.researchsquare.com/article/rs-757671/v1> |
| Asia | South Korea | <https://doi.org/10.5281/zenodo.5139512> | <https://www.researchsquare.com/article/rs-757722/v1> |
| Asia | Taiwan | <https://doi.org/10.5281/zenodo.5139520> | <https://www.researchsquare.com/article/rs-757733/v1> |
| Asia | Thailand | <https://doi.org/10.5281/zenodo.5139498> | <https://www.researchsquare.com/article/rs-757735/v1> |
| Asia | Vietnam | <https://doi.org/10.5281/zenodo.5139527> | <https://www.researchsquare.com/article/rs-757746/v1> |
| Asia | Papua New Guinea | <https://doi.org/10.5281/zenodo.5139488> | <https://www.researchsquare.com/article/rs-757653/v1> |
| South America | Argentina | <https://doi.org/10.5281/zenodo.5498081> | <https://www.researchsquare.com/article/rs-893102/v1> |
| South America | Bolivia | <https://doi.org/10.5281/zenodo.5498083> | <https://www.researchsquare.com/article/rs-893267/v1> |
| South America | Brazil | <https://doi.org/10.5281/zenodo.5498085> | <https://www.researchsquare.com/article/rs-893535/v1> |
| South America | Chile | <https://doi.org/10.5281/zenodo.5498087> | <https://www.researchsquare.com/article/rs-893607/v1> |
| South America | Colombia | <https://doi.org/10.5281/zenodo.5498091> | <https://www.researchsquare.com/article/rs-893706/v1> |
| South America | Ecuador | <https://doi.org/10.5281/zenodo.5498093> | <https://www.researchsquare.com/article/rs-893779/v1> |
| South America | Paraguay | <https://doi.org/10.5281/zenodo.5498099> | <https://www.researchsquare.com/article/rs-895567/v1> |
| South America | Peru | <https://doi.org/10.5281/zenodo.5498101> | <https://www.researchsquare.com/article/rs-895579/v1> |
| South America | Uruguay | <https://doi.org/10.5281/zenodo.5498103> | <https://www.researchsquare.com/article/rs-895585/v1> |
| South America | Venezuela | <https://doi.org/10.5281/zenodo.5498105> | <https://www.researchsquare.com/article/rs-895585/v1> |
