# Supplementary material for: Designing a zero-order energy transition model: How to create a new Starter Data Kit
Source: MethodsX. 2023 Mar 12;10:102120. doi: 10.1016/j.mex.2023.102120 (PMC10050781; doi:10.1016/j.mex.2023.102120)
Supplement: Supplementary file 2 [file mmc2.docx]

**Designing a zero-order energy transition model: a guide for creating a Starter Data Kit**

| **Title** | Designing a zero-order energy transition model: how to create a new Starter Data Kit |
| --- | --- |
| **Authors** | Carla Cannone* [1,2], Lucy Allington [1], Karla Cervantes Barron [3], Flora Charbonnier [4], Miriam Zachau Walker [4], Claire Halloran [4], Rudolf Yeganyan [1,2], Naomi Tan [1,2], Jonathan M Cullen [3], John Harrison [1], Long Seng To [1] and Mark Howells [1,2]. |
| **Affiliations** | 1. Centre for Sustainable Transitions: Energy, Environment & Resilience (STEER), Loughborough University, United Kingdom  2. Imperial College London, United Kingdom  3. University of Cambridge, United Kingdom  4. University of Oxford, United Kingdom |
| **Corresponding Author's email address** | C.Cannone@lboro.ac.uk |
| **Keywords** | Energy System Modelling  Data Collection Tool  OSeMOSYS  clicSAND  U4RIA |
| **Direct Submission or Co-Submission**  *Co-submissions are papers that have been submitted alongside an original research paper accepted for publication by another Elsevier journal* | *Co-Submission* |

**ABSTRACT**

The Paris Agreement was signed by 192 Parties, who committed to reducing emissions. Reaching such commitments by developing national decarbonisation strategies requires significant analyses and investment. Analyses for such strategies are often delayed due to a lack of accurate and up-to-date data for creating energy transition models. The Starter Data Kits address this issue by providing open-source, zero-level country datasets to accelerate the energy planning process. There is a strong demand for replicating the process of creating Starter Data Kits because they are currently only available for 69 countries in Africa, Asia, and South America. Using an African country as an example, this paper presents the methodology to create a Starter Data Kit from data collection to the creation of tool-agnostic data repositories. The paper illustrates the steps involved, provides additional information for conducting similar work in Asia and South America, and highlights the limitations of the current version of the Starter Data Kits. Future development is proposed to expand the datasets, including new and more accurate data and new energy sectors.

**SPECIFICATIONS TABLE**

| **Subject Area** | Energy |
| --- | --- |
| **More specific subject area** | Energy System Modelling |
| **Method name** | Data Collection and Manipulation Method for Starter Data Kits models |
| **Name and reference of original method** | Not applicable |
| **Resource availability** | Annex A - Links to Zenodo Repositories  Annex B - Methodology for Asian and South American Regions  Annex C - Main Boxes, Tables and Useful Files |

**ANNEX B**

For Asia, efficiencies of power plants and stoves were taken from the IRENA ASEAN REMap report [1]. For South America. Efficiencies of transport and heating technologies in all countries were taken from Terpilowski Gill (2020) Decarbonising the Laotian Energy System [2].

### **Technology Costs – Developing Asia**

Check if the country is a net importer/exporter/neutral for gas and coal and select the correct fuel prices from the Asian support file [3]. Costs of refinery technologies were taken from the TEMBA report [4]. Costs of power plants were mostly taken from the IRENA ASEAN REMap Report [5] – the cost, from IRENA report, for 'Oil' was used for Light Fuel Oil (LFO) plants. Then this was increased by 12% to estimate costs for High Fuel Oil (HFO) plants based on the relationship between LFO and HFO technology costs in the IRENA report used for Africa. The same was done for gas, where the IRENA ASEAN REMap value for gas was used for CCGTs and reduced by 21% to estimate OCGT costs. The costs of renewables were estimated from IRENA (2020) Renewable Power Generation Costs in 2019 [6], using the average values for all Asian countries considered, and the IRENA ASEAN ReMap report, with the cost reduction trends from the report used for Africa applied. Costs of transmission and distribution were estimated based on this ERIA report [7]. Generic costs of transport and heating technologies were taken from Terpilowski Gill (2020) Decarbonising the Laotian Energy System [2]. The costs of energy efficiency technologies were estimated based on the costs of coal power plants – see example calculations in the main text. Costs of stoves were estimated from the IRENA ASEAN REMap Report and IRENA (2017) Biogas for Domestic Cooking [8]. Costs of renewables with storage were estimated by combining the standard cost from the IRENA reports with an estimated storage cost based on the NREL 2020 Annual Technology Baseline [9] – see example calculations in the main text. Fuel prices were taken from the APEC 7^th^ Annual Energy Outlook [10]. The international biomass price was estimated from a report by Argus Media [11]a, and the domestic biomass price was estimated from an ERIA report [7]. More detail on costs can be found in the Base Data Preparation file for Asia.

### **Technology Costs – South America**

Costs of refinery technologies were taken from the TEMBA report [4]. Costs of transmission and distribution technologies from SAMBA dataset [12]. Costs for renewables taken from SAMBA dataset from 2013 to 2058 – linear change between data points (2013 and 2058). Then constant from 2058 until the end. The cost of CSP with Storage was estimated from the cost of CSP without storage (from SAMBA Dataset) - it was applied a percentage increase taken from IRENA ACEC (Africa Clean Energy Corridor)[13]. The cost of Medium Hydropower plants (10-100MW) and Solar PV (Distributed with Storage) was taken from IRENA ACEC Report, as no data was available in SAMBA. The costs of Off-grid Hydropower plants were assumed to be the same as Small Hydropower Plants. Costs for fossil power plants were taken from SAMBA Dataset from 2013 and 2058, then constant from 2058 until the end. The cost of the Coal Power Plant was assumed to be double the capital cost of the Gas Power Plant (CCGT). Therefore, the coal price from SAMBA Dataset was reduced to align with more recent reports. The Light Fuel Oil Power Plant cost was taken from IRENA (2018) Planning & Prospects for Renewable Power: West Africa [14] as no data were available in SAMBA Dataset. Generic costs of transport and heating technologies were taken from Terpilowski Gill (2020) Decarbonising the Laotian Energy System [2]. Price of Biomass Extraction was taken from Ricardo Energy & Environment – Global Biomass Markets [15]. Price of Coal Extraction was taken from SAMBA Dataset, assuming a linear growth between data points, the growth rate between 2020-2030 is continued to 2040, then the price is assumed constant to 2071. Prices of Natural Gas Extraction were taken from SAMBA Dataset – average value was used if no country-specific data were available). More detail on costs can be found in the Base Data Preparation file for South America. All the other costs were calculated as done in Africa.

| **Box 5. Doing country-specific Data Collection and Manipulation for Asia** **Country Selection** To work on the desired country, the user must create a copy of the Africa Base Data Collection File (without opening the file) for the selected country in a Data Preparation and Manipulation folder. Name the file: **New *countryname* Data Collection**. Then, the file must be opened. This step, along all those remaining in Section 1.2.1 may need some time to load.  The links to other files must be updated so the linked data is available and up to date. Figure 2 shows how to update the links by clicking on the *Data* tab*,* followed by clicking on *Edit Links*.  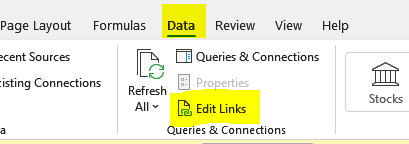  Figure 1: Updating links on Excel spreadsheet of the Base Data Collection File.  The menu with the different linked worksheets should be displayed, where each source must be changed. Figure 3 shows a selected source, which can be changed by clicking on *Change Source*. This will open an explorer tab which allows the user to navigate to the folder with all the capacity factor and residual capacity data. The appropriate file can then be selected.  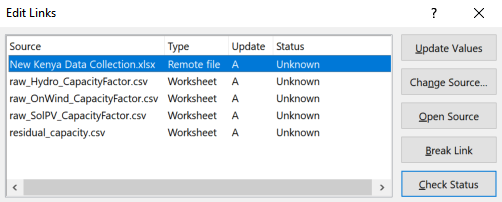  Figure 2: Changing the source of the different spreadsheets linked to the Base Data Collection File according to the user file paths.  Once all the links are working, the user can select the country from the drop-down menu in Tab 1. Model Initiation.  After the relevant data for the country has been selected, the user can break the links to the different worksheets updated earlier (instructions a–b) by clicking on the option of *Break Link*. This should make the spreadsheet work much faster. However, the user must be careful to select the correct values to avoid returning to the spreadsheet to change the country at a later stage, which would need linking the data again by rewriting the formulae- a long and error-prone step for less experienced users. **2. Power transmission and distribution output activity ratios** Use values from Asia data sheet which calculates Output Activity ratios based on the Index Mundi Website [16], assuming combined losses reach 5% in 2050. **3. PV, onshore & offshore wind and hydro capacity factors** This is automated apart from offshore wind. Check data for the selected country is inserted in the Raw PV/Onshore Wind/Hydro Capacity Factors tabs (not the (auto) tabs) and that output capacity factors for these technologies are in the output capacity factors tab. For offshore wind, take the average offshore capacity factor from the NREL Global Offshore Capacity Factor data [3] and insert it into the 3.6 Capacity Factor Calculation tab for offshore wind (cells E36–L36). Time zones are set up to calculate profiles for countries that are UTC +8, adjust this if the country is in a different timezone – change for PV and wind CFs and the demand profile.* **4. Off-grid capacity** Copy and paste the Cumulative/Additions Label for off-grid hydropower and off-grid solar PV for the country from the **IRENA Installed Capacities data.xlsx** (f[rom Cannone](https://zenodo.org/record/6142375) at al. [17]) into the Off-Grid Capacity tab. Note: if the IRENA file has the country but not the relevant off-grid technologies, the values can be assumed to be zero, and no user action is required.   - In the IRENA datasheet, ensure the 'Type' column (column AC) is filtered to '**off-grid.**' - Filter to the selected **country** in the 'IRENA Menu' column (column A) - In the '**Sub-technology**' column, filter to Solar Photovoltaic - Order by Years and then copy the 'Cumulative/Additions Label' column (column AL) into tab **3.7 Off-Grid Capacity**, cells C5–C24 – if there is no data for a year, then put 0, ensure the years match up with the years in column A - Repeat the process but filter out for Renewable Hydropower as the sub-technology, copying the Cumulative/Additions Label column into cells B5–B24 in the tab 3.7 Off-Grid Capacity   Check that the residual capacity rows for PWRSOL002 and PWRHYD004 (rows 920 and 1085) are updated in the **3.7 ResCap data (auto)** tab. If not in the IRENA list, the **TEMBA_21_10_Refer** file can be used. Go to the residual capacity tab:   - Copy the rows in the residual capacity tab for XXSOV1F01X and XXSOV2F01X from cell B to cell BE. If 0 in all years, no user action is required. - Paste those rows into tab 3.7 ResCap data (auto) tab in rows 1065 and 1066 (PWRSOL002), starting in column I (to the right of the yellow cells). - Ensure that the orange Residual Capacity row for PWRSOL002 (row 1085) is updated if adding values. - Off-grid hydropower is excluded in this case since TEMBA does not include this technology.  **5. On-grid residual capacity** This step relies on linked sources, so filling in some fields is done automatically. Check that on-grid residual capacity data for the selected country is in the 3.7 ResCap data (auto) tab. Focus on checking between rows 724–1197 as this is where the power plant technologies are (all begin with PWR).  Find the raw on-grid residual capacity data for the selected country in the file **residual capacity.csv** from Brinkerink and Deane [18] and check that there are no more than 11 power plants of each type for the selected country, as this is the maximum number permitted by the spreadsheet. If there are more than 11 rows, extra rows can be manually added, and extra data copied directly from the CSV file.  A few manual adjustments are needed:   - For hydropower (rows 852–920), move any plants in the PWRHYD001 group to PWRHYD002 if they have a capacity between 0.01–0.1 GW and to PWRHYD003 if less than 0.01 GW (capacity is in column H). To move a plant, copy the entire row for that plant from column E to column AR, and paste it in the corresponding cells of the intended technology group. - For oil power plants (rows 985–1016), all residual capacity is automatically inserted as PWROHC001 (LFO plant). However, to find more information, it can be done online research of the Power Plant's name, and any plants that do not belong to the right category should be moved to PWROHC002 (HFO gas turbine) using the method above. If no information can be found, leave the plant where it is. - The same should be done for gas power plants (rows 921–952), moving plants from PWRNGS001 (CCGT) to PWRNGS002 (OCGT) if it can be found out that the plant is an OCGT. If no reliable data is found, make no changes. Useful sites for checking the type of power plant are [19] [20]; these can be searched by power plant name or by country and cover both oil and gas.  **6. Refinery and Transmission & Distribution residual capacity** It was assumed that the transmission and distribution residual capacity is equal to the total installed power generation capacity in GW from the res cap csv. Note the PWR technologies (rows 724–1197 in tab 3.7 ResCap data (auto)) that do not have any residual capacity in the country, as this is needed for Step 7. Do not include the following technologies: PWRTRN, PWRDIST, PWRTRNIMP, PWRTRNEXP. Repeat these steps for the refinery technologies: UPSREF001 and UPSREF002 (rows 3166 to end) (XXCRUDRE1X & XXCRUDRE2X in TEMBA, respectively). **Step 8 - Capacity Constraints** In the 3.8 Capacity & Inv Constraints tab, set the **Total Annual Max Capacity Investment** for 2015–2020 to 0 (column E to column J) for power generation and refinery technologies that have 0 residual capacity in the country, using the list made in Step 7. Do not do this for the following technologies: PWRTRN (row 439), PWRDIST (row 440), PWRTRNIMP (row 437), PWRTRNEXP (row 443).  If the country has no offshore wind potential, put 0 for the **Total Annual Max Capacity** for offshore wind all years (PWRWND002, row 37, 0 should be input from column E to BH). Highlight the rows where these constraints have been added in green, as they will be pasted to SAND later. **7. Demands** Copy and paste the listed demands for your country into rows 3–6 in tab 4.1 Raw APEC & IEA Demand data from the APEC 7^th^ Annual Energy Outlook datasheet. Check rows 7–11 in the 4. TEMBA Demands Data tab have been filled in with data (rows 7–11, columns C–BF). Make sure this process is done in the **4. TEMBA Demands Data tab** (**not** the 4. TEMBA Demands Data (auto) tab).  For IEA countries, insert the final consumption (in PJ; convert from TJ if needed) for each fuel in each sector in the country for 2015–2018 into the table (rows 15–30, columns B to E) in the 4. TEMBA Demands Data tab from the IEA Sankey Diagram [21], marking 0 if there is no consumption for that fuel/sector. Key points:   - For this step, ensure the 'Final Consumption' option is selected rather than 'Balance' in the navigation pane on the left of the IEA website - Ensure the unit is changed to PJ/TJ at the top of the diagram, and be sure to convert to PJ if it is in TJ (divide by 1,000) - The consumption in each sector can be seen by clicking on the sector on the diagram, which opens a pie chart - Change the year by dragging the slide along the bottom   For non-IEA countries:   - Find the United Nations Energy Balance for the selected country on the UN website [22] (PDFs for groups of countries in alphabetical order). - For these countries, insert data in the 2018 and 2017 columns of the table in the 4. TEMBA demands data tab (columns D and E, rows 15–30). The UN energy balances are in TJ; so divide by 1,000 to convert to PJ when inserting data. The top section of the UN energy balance is usually marked as 2018, then 2017 data are below – but check this for the selected country. - Look at the data in the 'Final energy consumption' sections of the energy balance for 2017 and 2018. For industry, use the values for 'Manufacturing, const, mining'. For transport use the values for 'Transport'. For commerce use the values 'Commerce and public services’. For residential use the values for 'Households'. Use 'All Oil' for oil products; sum the values for 'Primary biofuels/Waste' and 'Charcoal' for biofuels and waste. - Leave the columns for 2015 and 2016 (columns B and C) blank, so they will not be considered in the average calculated in column F.   Demands will then be automatically calculated: check in the 4.1 Accumulated Annual Demand tab that the rows for TRAMCY, TRACAR, TRABUS, INDHEH, INDHEL, RESCKN, COMHEL, and RESHEL have been filled in (shaded in green, between rows 19–29), and in the 4.2 Specified Annual Demand tab that the rows for INDELC, RESELC, and COMELC (rows 22, 25, & 27) have been filled in (shaded in green). These demands consider the input activity ratios of the technologies used to deliver them; for example, the demand for motorcycles is initially calculated in terms of oil demand based on the input data, and this is then converted into the final energy demand for motorcycles considering the efficiency of the oil motorcycle technology.  **8. Electricity demand profile**  Copy and paste the hourly electricity demand profile for the selected country from the PLEXOS All Demand UTC 2015.tab dataset downloadable from Brinkerink and Deane [18] into the 4.2 Elc Demand Profile Raw Data tab.   - In PLEXOS the countries are along the tab (countries organized in columns), with the region code (AF for Africa), followed by the country code), copy the whole column starting from row 2 to row 8761. - Paste the column into tab **4.2 Elc Demand Profile Raw Data** starting in cell B4 (marked in yellow).   Go to the **4.2 Specified Dem Profile Calc** tab and to the rows for RESELC, COMELC, and INDELC (rows 21, 24, and 26). Adjust the value in column L (Bennet Factor) until the value in column M is exactly equal to 1. Only small adjustments are needed: e.g., if the value in column M is 1.007, first try adjusting the value in column L to 0.98, then make further small adjustments if needed. Check that Specified Demand Profiles have been calculated for RESELC, COMELEC, and INDELC in the 4.2 Specified Demand Profile Output tab (columns W, Z, AB). **9. Import & Export activity limits** For IEA countries: Insert the amounts of imported and exported electricity (PJ) from the IEA Sankey diagram [21] for the country for 2015–2018 into the TotalTechnologyAnnualActivityUpperLimit rows for PWRTRNIMP (row 238) and PWRTRNEXP (row 244) in tab 5.1 Activity in columns F (2015) to I (2018). Columns beyond column I are automatically calculated based on the values entered in columns F to I. Important points:   - For this Step ensure the **'Energy Balance'** option is selected in the left-hand navigation pane on the IEA Sankey website. - Ensure that the unit is set to PJ/TJ as in Step 8, and carry out unit conversions if needed. - Data can also be obtained from the IEA's energy balance tables [21]. - If there is no data, set to 0.   For non-IEA countries: open the UN energy balance for the selected country used in the earlier demands step. UN data are in TJ, which must be divided by 1,000 to convert to PJ. Go to tab 5.1 Activity. Insert the amounts of imported and exported electricity from the energy balance in 2017 and 2018 into the TotalTechnologyAnnualActivityUpperLimit rows for PWRTRNIMP (row 238) and PWRTRNEXP (row 244) in column H for 2017 and column I for 2018. Electricity imports and exports are found in the UN energy balance in the top section for each year in the rows for 'Imports' and 'Exports' under 'Electricity'. Columns beyond column I are automatically calculated based on the values entered in columns F to I. Important points:   - The values for 2017 must be inserted into the columns for 2015 and 2016 (columns F and G) – it is assumed that imports & exports remain similar across years. - Do not include the minus sign found before the values for exports in the UN energy balance data. - Remember that the UN data are in TJ and must be divided by 1,000 to convert to PJ.  **10. Renewable and fossil resources** Insert the estimated renewable energy potentials in the country into the table in the tab Data in Brief Tables 8 & 9 from the sources indicated in the table below. Some notes:   - Brunei: sources in table plus 70-80Mw hydro from <https://www.reeep.org/brunei-darussalam-2012> - Indonesia: sources in table plus 41.426GW hydro (+450MW SHP if not in world shp report), 27.15GW geothermal from RBAP SE4ALL report (available at <https://www.asia-pacific.undp.org/content/rbap/en/home/library/climate-and-disaster-resilience/APRC-EE-2013-SE4ALL.html>) - Papua New Guinea: 150MW wind, 4GW hydro (+5.7Mw SHP), 0.8GW geothermal from RBAP SE4ALL report - Malaysia: sources below plus 0.49GW hydro from RBAP SEA4ALL report - Myanmar: 40.4GW hydro and 4GW wind from <https://accept.aseanenergy.org/wp-content/uploads/2020/01/A-Review-of-RE-and-NDCs-in-ASEAN.pdf> - Philipines: sources below plus 4GW geothermal and 10.5GW hydro from <https://accept.aseanenergy.org/wp-content/uploads/2020/01/A-Review-of-RE-and-NDCs-in-ASEAN.pdf> - Thailand: sources below plus 4.542GW hydro (+50MW SHP) from RBAP SE4ALL report - Vietnam: sources below plus 3.8GW hydro (+2.887GW small hydro) and 10GW pumped hydro and 0.4GW geothermal from RBAP SE4ALL report   Table 1: Data sources for Hydropower potential in Asian countries   \| Country \| Small Hydro \| PV and Wind \| Solar Resource \| \| --- \| --- \| --- \| --- \| \| Brunei, Indonesia, Malaysia, Philippines, Singapore, Thailand, Vietnam \| World Small Hydropower Development Report \| [Exploring RE opportunities in select SE Asian countries 2019](https://www.nrel.gov/docs/fy19osti/71814.pdf) report (use values in table at start with LCOE <$150/MWh) \| NREL datasets (see [here](https://docs.google.com/spreadsheets/d/1MalTV4LUKf6-Qw_sHJh-igRsdn4aoCptqEnEBpabxn4/edit?usp=sharing)) \|   If the user is working on a new country, it is recommended to first check the sources in the table above for potentials and then fill any gaps with country-specific sources. The NREL wind capacity factor datasets (referenced [here](https://docs.google.com/spreadsheets/d/1MalTV4LUKf6-Qw_sHJh-igRsdn4aoCptqEnEBpabxn4/edit?usp=sharing)) can also be used to estimate wind resources. Insert the estimated fossil fuel reserves in the country into the table in the tab Data in Brief Tables 8 & 9 from the table on page 88 of the TEMBA report ([here](https://publications.jrc.ec.europa.eu/repository/bitstream/JRC118432/jrc118432_jrc118432_reviewed_by_ipo.pdf)). If there is a dash, assume 0. If the country is not in the table, this means it will be assumed 0 domestic reserves so insert 0 for coal, gas and oil.  Check that total technology model period activity upper limits have been added for MINOIL, MINNGS and MINCOA in the 5.1 Activity tab (row 601, 605, 611), and that total annual max capacity limits have been updated for PWRGEO (row 24) and PWRHYD001-004 (rows 33, 34, 35) in the tab 3.8 Capacity & Inv Constraints if applicable. |
| --- | --- | --- | --- | --- | --- | --- | --- | --- |

##

| **Box 6. Doing country-specific Data Collection and Manipulation for South America** **Country Selection** To work on the desired country, the user must create a copy of the Africa Base Data Collection File (without opening the file) for the selected country in a Data Preparation and Manipulation folder. Name the file: **New *countryname* Data Collection**. Then, the file must be opened. This step, along all those remaining in Section 1.2.1 may need some time to load.  The links to other files must be updated so the linked data is available and up to date. Figure 2 shows how to update the links by clicking on the *Data* tab*,* followed by clicking on *Edit Links*.  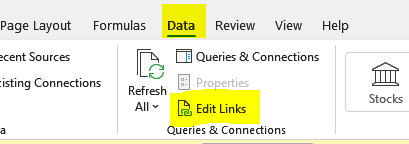  Figure 3: Updating links on Excel spreadsheet of the Base Data Collection File.  The menu with the different linked worksheets should be displayed, where each source must be changed. Figure 3 shows a selected source, which can be changed by clicking on *Change Source*. This will open an explorer tab which allows the user to navigate to the folder with all the capacity factor and residual capacity data. The appropriate file can then be selected.  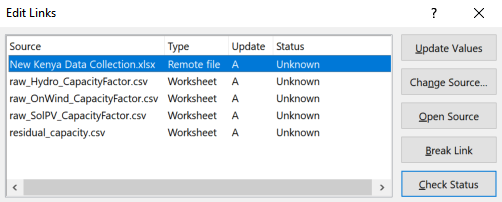  Figure 4: Changing the source of the different spreadsheets linked to the Base Data Collection File according to the user file paths.  Once all the links are working, the user can select the country from the drop-down menu in Tab 1. Model Initiation.  After the relevant data for the country has been selected, the user can break the links to the different worksheets updated earlier (instructions a–b) by clicking on the option of *Break Link*. This should make the spreadsheet work much faster. However, the user must be careful to select the correct values to avoid returning to the spreadsheet to change the country at a later stage, which would need linking the data again by rewriting the formulae- a long and error-prone step for less experienced users. **Step 2 Adjust for the Time Zone for PV, wind and specified demand as done for Asia.** **Step 3 - Power transmission and distribution output activity ratios** Copy the output activity ratio rows for power transmission and distribution from the Data_Reference_SAMBA dataset to the rows in the 3.3 Output Activity Ratios tab. In SAMBA, in LossesTrans&Distr tab there is one table for transmission losses and one for distribution per each country.  In SAMBA LossesTrans&Distr tab, copy the entire row of your country from Column D to column BG and paste into row 41 of tab 3.3 Output Activity Ratios, starting in column F. For Brazil use the Brazil Average row 7. In SAMBA LossesTrans&Distr tab, copy the entire row for your country from column D to column BG and paste into row 42 of tab 3.2 Output Activity Ratios, starting in column F. For Brazil use the Brazil Average row 24. **Step 4 - PV, onshore wind and hydro capacity factors** This is automated. Check data for your country is inserted in the Raw PV/Onshore Wind/Hydro/Offshore Wind CFs tabs (not the (auto) tabs) and that output capacity factors for these technologies are in the output capacity factors tab. Offshore wind capacity factors will come from the NREL dataset. Copy and paste the average capacity factor (row L, cells 28–37, highlighted in green) for the country from [this spreadsheet](https://lunet.sharepoint.com/:x:/r/sites/CCG-grp/Shared%20Documents/SHARED-%20CCG%20partners/OA4_Platform/Starter%20Kit/Data%20Preparation%20%26%20Manipulation/South%20America%20Offshore%20Wind%20CFs.xlsx?d=wf1852aa3d6274c938989a206fe8d4ff3&csf=1&web=1&e=67fCMW) into the 3.6 Capacity Factor Calculation tab for PWRWND002 for every time slice. **Step 5 – Refinery Residual Capacity** Copy and paste the refinery capacity in kb/d from the starter kits – list of countries spreadsheet [23] into the 3.7 Refinery ResCap tab. This will automatically be converted to the residual capacity output for UPSREF001. (The primary source for the refinery capacity is [McKinsey South American refineries](https://www.mckinseyenergyinsights.com/resources/refinery-reference-desk/latin-american-refineries/) [24]). **Step 6 - Off-grid capacity** Copy and paste the Cumulative/Additions Label for off-grid hydropower and off-grid solar PV for the country from the **IRENA Installed Capacities data.xlsx** (f[rom Cannone](https://zenodo.org/record/6142375) at al. [17]) into the Off-Grid Capacity tab. Note: if the IRENA file has the country but not the relevant off-grid technologies, the values can be assumed to be zero, and no user action is required.   - In the IRENA datasheet, ensure the 'Type' column (column AC) is filtered to '**off-grid.**' - Filter to the selected **country** in the 'IRENA Menu' column (column A) - In the '**Sub-technology**' column, filter to Solar Photovoltaic - Order by Years and then copy the 'Cumulative/Additions Label' column (column AL) into tab **3.7 Off-Grid Capacity**, cells C5–C24 – if there is no data for a year, then put 0, ensure the years match up with the years in column A - Repeat the process but filter out for Renewable Hydropower as the sub-technology, copying the Cumulative/Additions Label column into cells B5–B24 in the tab 3.7 Off-Grid Capacity   Check that the residual capacity rows for PWRSOL002 and PWRHYD004 (rows 920 and 1085) are updated in the **3.7 ResCap data (auto)** tab. If not in the IRENA list, the **TEMBA_21_10_Refer** file can be used. Go to the residual capacity tab:   - Copy the rows in the residual capacity tab for XXSOV1F01X and XXSOV2F01X from cell B to cell BE. If 0 in all years, no user action is required. - Paste those rows into tab 3.7 ResCap data (auto) tab in rows 1065 and 1066 (PWRSOL002), starting in column I (to the right of the yellow cells). - Ensure that the orange Residual Capacity row for PWRSOL002 (row 1085) is updated if adding values. - Off-grid hydropower is excluded in this case since TEMBA does not include this technology.  **Step 7 - On-grid residual capacity** This is automated. Check that on-grid residual capacity data for your country is in the 3.7 ResCap data (auto) tab. Focus on checking between rows 724–1197 as this is where the power plant technologies are (all begin with PWR). The raw on-grid residual capacity data for your country can be found in a csv file [here](https://lunet.sharepoint.com/:f:/r/sites/CCG-grp/Shared%20Documents/SHARED-%20CCG%20partners/OA4_Platform/Starter%20Kit/Data%20Preparation%20%26%20Manipulation/data/residual_capacity?csf=1&web=1&e=bq6tOH) - check that there are no more than 11 power plants of each type in your country as this is the maximum number permitted by the spreadsheet. A few manual adjustments are needed:   - For hydropower (rows 852–920), move any plants in the PWRHYD001 group to PWRHYD002 if they have a capacity between 0.01–0.1 GW and to PWRHYD003 if less than 0.01 GW (capacity is in column H). To move a plant, copy the entire row for that plant from column E to column AR, and paste it in the corresponding cells of the intended technology group. - For oil power plants (rows 985–1016), all residual capacity is automatically inserted as PWROHC001 (LFO plant). However, to find more information, it can be done online research of the Power Plant's name, and any plants that do not belong to the right category should be moved to PWROHC002 (HFO gas turbine) using the method above. If no information can be found, leave the plant where it is. - The same should be done for gas power plants (rows 921–952), moving plants from PWRNGS001 (CCGT) to PWRNGS002 (OCGT) if it can be found out that the plant is an OCGT. If no reliable data is found, make no changes. Useful sites for checking the type of power plant are [19] [20]; these can be searched by power plant name or by country and cover both oil and gas.  **Step 8 – Cooking Residual Capacity** Insert the proportions of each fuel used for cooking (as a decimal) in the country from the country profile [25]. This source gives estimates of the % of cooking done with each fuel. Count LPG and kerosene as oil. Cooking residual capacity will be automatically estimated once the accumulated annual demand for RESCKN is filled in. The user can copy and paste the res cap rows for the stove technologies into SAND files. If the country isn't in that source, just search online to estimate.  Important note: TotalTechnologyAnnualActivityUpperLimit for DEMRESCKNBIO will be automatically calculated using the % of cooking that is done with biomass currently and the RESCKN demand for each year – make sure to copy this into SAND. **Step 9 - Transmission & Distribution residual capacity** For transmission and distribution, it was assumed that the residual capacity is equal to the installed on-grid residual power plant capacity. Go to the res_cap CSV file for the country and work out the sum of all the capacities. Then insert this value for all years for PWRTRN and PWRDIST residual capacity. Make a note of the PWR technologies (rows 724-1197 in tab 3.7 ResCap data (auto)) that do not have any residual capacity in the country; it will be used later in Step 7. Do not include the following technologies: PWRTRN, PWRDIST, PWRTRNIMP, PWRTRNEXP. Additionally, do this for the refinery technologies: UPSREF001 and UPSREF002 (rows 3166 to end). **Step 10 - Capacity Constraints** In the 3.8 Capacity & Inv Constraints tab, set the **Total Annual Max Capacity Investment** for 2015–2020 to 0 (column E to column J) for power generation and refinery technologies that have 0 residual capacity in the country, using the list made in Step 7. Do not do this for the following technologies: PWRTRN (row 439), PWRDIST (row 440), PWRTRNIMP (row 437), PWRTRNEXP (row 443).  If the country has no offshore wind potential, put 0 for the **Total Annual Max Capacity** for offshore wind all years (PWRWND002, row 37, 0 should be input from column E to BH). Highlight the rows where these constraints have been added in green, as they will be pasted to SAND later. **Step 11 - Demands** Regional demands taken from the Energy Outlook of South America and the Caribbean 2019 [26]. This step relies on linked sources, so filling in some fields is done automatically, but only partially. Check rows 7–11 in the 4. TEMBA Demands Data tab have been filled in with data (rows 7–11, columns C–BF). Make sure this process is done in the **4. TEMBA Demands Data tab** (**not** the 4. TEMBA Demands Data (auto) tab).  For IEA countries, insert the final consumption (in PJ; convert from TJ if needed) for each fuel in each sector in the country for 2015–2018 into the table (rows 15–30, columns B to E) in the 4. TEMBA Demands Data tab from the IEA Sankey Diagram [21], marking 0 if there is no consumption for that fuel/sector. Key points:   - For this step, ensure the 'Final Consumption' option is selected rather than 'Balance' in the navigation pane on the left of the IEA website - Ensure the unit is changed to PJ/TJ at the top of the diagram, and be sure to convert to PJ if it is in TJ (divide by 1,000) - The consumption in each sector can be seen by clicking on the sector on the diagram, which opens a pie chart - Change the year by dragging the slide along the bottom   For non-IEA countries:   - Find the United Nations Energy Balance for the selected country on the UN website [22] (PDFs for groups of countries in alphabetical order). - For these countries, insert data in the 2018 and 2017 columns of the table in the 4. TEMBA demands data tab (columns D and E, rows 15–30). The UN energy balances are in TJ; so divide by 1,000 to convert to PJ when inserting data. The top section of the UN energy balance is usually marked as 2018, then 2017 data are below – but check this for the selected country. - Look at the data in the 'Final energy consumption' sections of the energy balance for 2017 and 2018. For industry, use the values for 'Manufacturing, const, mining'. For transport use the values for 'Transport'. For commerce use the values 'Commerce and public services’. For residential use the values for 'Households'. Use 'All Oil' for oil products; sum the values for 'Primary biofuels/Waste' and 'Charcoal' for biofuels and waste. - Leave the columns for 2015 and 2016 (columns B and C) blank, so they will not be considered in the average calculated in column F.   Demands will then be automatically calculated: check in the 4.1 Accumulated Annual Demand tab that the rows for TRAMCY, TRACAR, TRABUS, INDHEH, INDHEL, RESCKN, COMHEL, and RESHEL have been filled in (shaded in green, between rows 19–29), and in the 4.2 Specified Annual Demand tab that the rows for INDELC, RESELC, and COMELC (rows 22, 25, & 27) have been filled in (shaded in green). These demands consider the input activity ratios of the technologies used to deliver them; for example, the demand for motorcycles is initially calculated in terms of oil demand based on the input data, and this is then converted into the final energy demand for motorcycles considering the efficiency of the oil motorcycle technology. **Step 12 - Electricity demand profile** Copy and paste the hourly electricity demand profile for the selected country from the PLEXOS All Demand UTC 2015.tab dataset downloadable from Brinkerink and Deane [18] into the 4.2 Elc Demand Profile Raw Data tab.   - In PLEXOS the countries are along the tab (countries organized in columns), with the region code (SA for South America), followed by the country code, copy the whole column starting from row 2 to row 8761. - Paste the column into tab **4.2 Elc Demand Profile Raw Data** starting in cell B4 (marked in yellow).   Go to the **4.2 Specified Dem Profile Calc** tab and to the rows for RESELC, COMELC, and INDELC (rows 21, 24, and 26). Adjust the value in column L (Bennet Factor) until the value in column M is exactly equal to 1. Only small adjustments are needed: e.g., if the value in column M is 1.007, first try adjusting the value in column L to 0.98, then make further small adjustments if needed. Check that Specified Demand Profiles have been calculated for RESELC, COMELEC, and INDELC in the 4.2 Specified Demand Profile Output tab (columns W, Z, AB). **Step 13 - Import & Export activity limits** For IEA countries: Insert the amounts of imported and exported electricity (PJ) from the IEA Sankey diagram [21] for the country for 2015–2018 into the TotalTechnologyAnnualActivityUpperLimit rows for PWRTRNIMP (row 238) and PWRTRNEXP (row 244) in tab 5.1 Activity in columns F (2015) to I (2018). Columns beyond column I are automatically calculated based on the values entered in columns F to I. Important points:   - For this Step ensure the **'Energy Balance'** option is selected in the left-hand navigation pane on the IEA Sankey website. - Ensure that the unit is set to PJ/TJ as in Step 8, and carry out unit conversions if needed. - Data can also be obtained from the IEA's energy balance tables [21]. - If there is no data, set to 0.   For non-IEA countries: open the UN energy balance for the selected country used in the earlier demands step. UN data are in TJ, which must be divided by 1,000 to convert to PJ. Go to tab 5.1 Activity. Insert the amounts of imported and exported electricity from the energy balance in 2017 and 2018 into the TotalTechnologyAnnualActivityUpperLimit rows for PWRTRNIMP (row 238) and PWRTRNEXP (row 244) in column H for 2017 and column I for 2018. Electricity imports and exports are found in the UN energy balance in the top section for each year in the rows for 'Imports' and 'Exports' under 'Electricity'. Columns beyond column I are automatically calculated based on the values entered in columns F to I. Important points:   - The values for 2017 must be inserted into the columns for 2015 and 2016 (columns F and G) – it is assumed that imports & exports remain similar across years. - Do not include the minus sign found before the values for exports in the UN energy balance data. - Remember that the UN data are in TJ and must be divided by 1,000 to convert to PJ.  **Step 14 - Renewable and fossil resources** Insert the estimated renewable energy potentials in the country into the table in the tab Data in Brief Tables 8 & 9 from the sources indicated in the table below. I have made screenshots of the tables needed from each report to save the time of going through the reports.  Table 2: Data sources for Renewable resources in South American countries   \| Country \| Small Hydro \| Hydro & Geothermal (where applicable) \| PV, CSP, Wind \| \| --- \| --- \| --- \| --- \| \| Argentina, Bolivia, Brazil, Chile, Colombia, Ecuador, French Guiana, Guyana, Paraguay, Peru, Suriname, Uruguay, Venezuela \| World Small Hydropower Development Report  [27] \| Data_Reference_SAMBA substract the data for small hydropower potential [12] \| Solar resource and wind potential from this NREL dataset [28]. Copy and paste the 'Total' value (column J) from the Solar Resource tab [here](https://lunet.sharepoint.com/:x:/r/sites/CCG-grp/Shared%20Documents/SHARED-%20CCG%20partners/OA4_Platform/Starter%20Kit/Data%20Preparation%20%26%20Manipulation/South%20America%20Offshore%20Wind%20CFs,%20Wind%20%26%20Solar%20Potentials.xlsx?d=wf1852aa3d6274c938989a206fe8d4ff3&csf=1&web=1&e=4Wg6Mb) into the 'Solar Resource' row in the table. From the same spreadsheet, copy and paste the offshore and onshore wind potentials in TWh/yr into the relevant row in the table. \|   Important note: total technology annual activity upper limit for PWRWND002 will be automatically calculated based on the potential just added to the table – make sure to add that to SAND.  Insert the estimated fossil fuel reserves in the country into the table in the tab Data in Brief Tables 8 & 9 from the table from Data_Reference_SAMBA report. If there is a dash, assume 0. If the country is not in the table, this means it will be assumed 0 domestic reserves so insert 0 for coal and oil.  Coal -> Coal for Electricity Tab; Oil -> HF for Electricity. For natural gas reserves data were taken from [BP Statistical Review of World Energy 2019 \| 68th edition.](https://www.bp.com/content/dam/bp/business-sites/en/global/corporate/pdfs/energy-economics/statistical-review/bp-stats-review-2019-natural-gas.pdf)Check that total technology model period activity upper limits have been added for MINOIL, MINNGS and MINCOA in the 5.1 Activity tab (row 601, 605, 611), and that total annual max capacity limits have been updated for PWRGEO (row 24) and PWRHYD001-004 (rows 33, 34, 35) in the tab 3.8 Capacity & Inv Constraints if applicable. |
| --- | --- | --- | --- | --- | --- | --- | --- | --- |

**References**

[1] “A Renewable Energy Roadmap RENEWABLE ENERGY OUTLOOK FOR ASEAN A Renewable Energy Roadmap A Renewable Energy Roadmap,” Accessed: Feb. 25, 2022. [Online]. Available: www.irena.org/publications,.

[2] E. Terpilowski-Gill, “Decarbonising the Laotian energy system Imperial College London, 2020.,” Imperial College London, 2020.

[3] L. Allington, “Asia Data Support File,” Feb. 2022, doi: 10.5281/ZENODO.6311949.

[4] I. Pappis *et al.*, “Energy projections for African countries ,” 2019. doi: 10.2760/678700.

[5] IRENA and ASEAN Centre for Energy, “Renewable Energy Outlook for ASEAN,” Abu Dhabi, 2016.

[6] IRENA, “Renewable Power Generation Costs in 2019,” Abu Dhabi, 2020.

[7] Y. Li and Y. Chang, “Infrastructure Investments for Power Trade and Transmission in ASEAN+2: Costs, Benefits, Long-Term Contracts, and Prioritised Development,” 2014. Accessed: Feb. 28, 2022. [Online]. Available: http://www.adb.org/features/fast-facts-asean-infrastructure-fund.

[8] “Biogas for Domestic Cooking: Technology brief.” https://www.irena.org/publications/2017/Dec/Biogas-for-domestic-cooking-Technology-brief (accessed Feb. 28, 2022).

[9] NREL, “Annual Technology Baseline 2020 Data,” 2020.

[10] Asia-Pacific Economic Cooperation, “APEC Energy Demand and Supply Outlook 7th Edition,” 2019.

[11] Argus, “Argus Biomass Markets Weekly Biomass Market News and Analysis Issue 20-47,” 2020.

[12] G. N. P. de Moura, L. F. L. Legey, and M. Howells, “A Brazilian perspective of power systems integration using OSeMOSYS SAMBA – South America Model Base – and the bargaining power of neighbouring countries: A cooperative games approach,” *Energy Policy*, vol. 115, pp. 470–485, Apr. 2018, doi: 10.1016/j.enpol.2018.01.045.

[13] “Africa Clean Energy Corridor.” https://irena.org/cleanenergycorridors/Africa-Clean-Energy-Corridor (accessed Feb. 28, 2022).

[14] I. Renewable Energy Agency, “Planning and prospects for renewable power: WEST AFRICA 2018,” 2018, Accessed: Feb. 21, 2022. [Online]. Available: www.irena.org.

[15] P. Howes, J. Bates, A. Brown, R. Diaz-Chavez, S. Christie, and A. Bayley, “Global Biomass Markets Final Report,” 2018.

[16] “Countries ranked by Electric power transmission and distribution losses (% of output) - Asia.” https://www.indexmundi.com/facts/indicators/EG.ELC.LOSS.ZS/rankings/asia (accessed Feb. 28, 2022).

[17] C. Cannone *et al.*, “Starter Kits - Technology-specific data for Base SAND file,” *CCG Starter Kits Supporting Data and Scripts*, 2022. .

[18] M. Brinkerink and P. Deane, “PLEXOS-World 2015,” 2020.

[19] “Global Energy Monitor.” https://www.gem.wiki/Main_Page (accessed Feb. 21, 2022).

[20] “List of Gas PowerPlants - GEO.” http://globalenergyobservatory.org/list.php?db=PowerPlants&type=Gas (accessed Feb. 21, 2022).

[21] International Energy Agency, “IEA Sankey Diagram,” 2019. .

[22] “UNSD — Energy Statistics.” https://unstats.un.org/unsd/energystats/pubs/balance/ (accessed Jun. 03, 2020).

[23] C. Cannone *et al.*, “Starter Kit - List of Countries and Regions,” *CCG Starter Kits Supporting Data and Scripts*, 2022. .

[24] McKinsey, “McKinsey Refinery Reference Desk,” 2020. .

[25] “ALL CLEAN PVT LT | Clean Cooking Alliance.” https://cleancooking.org/sector-directory/all-clean-pvt-lt/ (accessed Feb. 28, 2022).

[26] OLADE, “Energy Outlook of Latin America and the Caribbean 2019,” 2019.

[27] United Nations, “World Small Hydropower Development Report 2019,” 2019.

[28] “Open Energy Data Initiative (OEDI).” https://data.openei.org/ (accessed Feb. 28, 2022).
