# Supplementary material for: Designing a zero-order energy transition model: How to create a new Starter Data Kit
Source: MethodsX. 2023 Mar 12;10:102120. doi: 10.1016/j.mex.2023.102120 (PMC10050781; doi:10.1016/j.mex.2023.102120)
Supplement: Supplementary file 3 [file mmc3.docx]

**Designing a zero-order energy transition model: a guide for creating a Starter Data Kit**

| **Title** | Designing a zero-order energy transition model: how to create a new Starter Data Kit |
| --- | --- |
| **Authors** | Carla Cannone* [1,2], Lucy Allington [1], Karla Cervantes Barron [3], Flora Charbonnier [4], Miriam Zachau Walker [4], Claire Halloran [4], Rudolf Yeganyan [1,2], Naomi Tan [1,2], Jonathan M Cullen [3], John Harrison [1], Long Seng To [1] and Mark Howells [1,2]. |
| **Affiliations** | 1. Centre for Sustainable Transitions: Energy, Environment & Resilience (STEER), Loughborough University, United Kingdom  2. Imperial College London, United Kingdom  3. University of Cambridge, United Kingdom  4. University of Oxford, United Kingdom |
| **Corresponding Author's email address** | C.Cannone@lboro.ac.uk |
| **Keywords** | Energy System Modelling  Data Collection Tool  OSeMOSYS  clicSAND  U4RIA |
| **Direct Submission or Co-Submission**  *Co-submissions are papers that have been submitted alongside an original research paper accepted for publication by another Elsevier journal* | *Co-Submission* |

**ABSTRACT**

The Paris Agreement was signed by 192 Parties, who committed to reducing emissions. Reaching such commitments by developing national decarbonisation strategies requires significant analyses and investment. Analyses for such strategies are often delayed due to a lack of accurate and up-to-date data for creating energy transition models. The Starter Data Kits address this issue by providing open-source, zero-level country datasets to accelerate the energy planning process. There is a strong demand for replicating the process of creating Starter Data Kits because they are currently only available for 69 countries in Africa, Asia, and South America. Using an African country as an example, this paper presents the methodology to create a Starter Data Kit from data collection to the creation of tool-agnostic data repositories. The paper illustrates the steps involved, provides additional information for conducting similar work in Asia and South America, and highlights the limitations of the current version of the Starter Data Kits. Future development is proposed to expand the datasets, including new and more accurate data and new energy sectors.

**SPECIFICATIONS TABLE**

| **Subject Area** | Energy |
| --- | --- |
| **More specific subject area** | Energy System Modelling |
| **Method name** | Data Collection and Manipulation Method for Starter Data Kits models |
| **Name and reference of original method** | Not applicable |
| **Resource availability** | Annex A - Links to Zenodo Repositories  Annex B - Methodology for Asian and South American Regions  Annex C - Main Boxes, Tables and Useful Files |

**Data Downloading Requirements**

The methodology described in this paper refers to different databases and scripts. For ease of use, all the databases and scripts needed are presented in Table1 . The reader is advised to download all relevant files for the region of interest to work on an example.

Table 1: List of databases and scripts needed to repeat this methodology

| Name | File(s) | Link | Source | Description |
| --- | --- | --- | --- | --- |
| CCG Starter Kits - Base SAND file for Africa | Africa_base_SAND.xlsm | <https://zenodo.org/record/6011229> | [1] | This file is the Base SAND file for Africa. |
| CCG Starter Kits - Base SAND file for South America | South_America_base_SAND.xlsx | <https://zenodo.org/record/6108078> | [2] | This file is the Base SAND file for South America. |
| CCG Starter Kits - Base SAND file for Asia | Asia_base_SAND.xlsx | <https://zenodo.org/record/6109104> | [3] | This file is the Base SAND file for Asia. |
| CCG Starter Kits - Base SAND file for Africa Coal and Natural Gas | Africa_COA_NGS_base_SAND.xlsm | <https://zenodo.org/record/6033764> | [4] | This file is the Base SAND file for Africa with coal and natural gas. |
| CCG Starter Kits - Base SAND file for Africa Natural Gas Scenario | Africa_NGS_base_SAND | <https://zenodo.org/record/6036135> | [5] | This file is the Base SAND file for Africa with natural gas. |
| CCG Starter Kits - Base SAND file for Asia – Coal and Natural Gas | Asia_COA_NGS_base_SAND | <https://zenodo.org/record/6036147> | [6] | This is the Base SAND file for Asia with coal and natural gas. |
| CCG Starter Kits - Base SAND file for South America – Coal and Natural Gas Scenario | South_America_COA_NGS_base_SAND | <https://zenodo.org/record/6036192> | [7] | This is the Base SAND file for South America with coal and natural gas. |
| CCG Starter Kits - Script for CSV Creation with Base SAND Data for Starter Kits | ccg_data_to_sand.py | <https://zenodo.org/record/6036155> | [8] | Script to create comma-separated-value (CSV) file for base SAND file from the data collection file. |
| CCG Starter Kit - List of Countries and Regions | Starter Kit - List of Countries and Regions | <https://zenodo.org/record/6036252> | [9] | Mapping of countries to their relevant region. |
| CCG Starter Kits - Technology-specific Data for Base SAND File | Several csv files | <https://zenodo.org/record/6142375> | [10] | These files contain the Capacity Factors and Residual Capacity values for all countries needed to fill in the base SAND file information. |
| CCG Starter Kits - Scripts for SAND Scenarios | CCG_SAND_Scenario_XXX.py | <https://zenodo.org/record/6136904> | [11] | These files contain scripts to run the scenarios used in the Starter Kits. Each script creates a CSV file with the parameters for a specific scenario (corresponding to the script name) for a country or list of countries. The scenarios included are Fossil Future (FF), Least-Cost (LC), and Net Zero (NZ). |

Box 1 shows the country-specific data collection and manipulation process in detail.

| **Box 1. Doing country-specific Data Collection and Manipulation** Country Selection If the user wish to expand an existing Starter Data Kit, the authors recommend to download the “New_Country_Name_Data Collection.xlsx” file available in each country Starter Data Kit repository on Zenodo (more info was provided in Section 5.1. of the main text). Alternatively, to work on a country currently not covered in the existing Starter Data Kits repository [12] the user must make a copy of the Africa Base Data Collection File (Africa_base_SAND.xlsm -without opening the file) for the selected country. Using the working directory of the user’s choice is fine, yet copying the file into a folder called Data Preparation and Manipulation is advised. Name the file: **New *countryname* Data Collection**. Then, the file must be opened. This step, along all those remaining in Section 1.2.1 may need some time to load.  The links to other files that the base SAND file contains must be updated so the linked data is available and up to date. Figure 1 shows how to update the links by clicking on the *Data* tab*,* followed by clicking on *Edit Links*.  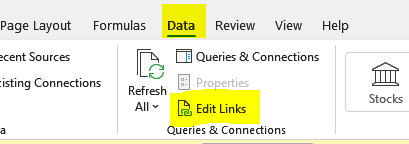  Figure 1: Updating links on Excel spreadsheet of the Base Data Collection File.  The menu with the different linked worksheets should be displayed, where each source must be changed. Figure 2 shows a selected source, which can be changed by clicking on *Change Source*. This will open an explorer tab which allows the user to navigate to the folder with all the capacity factor and residual capacity data. The appropriate file can then be selected.  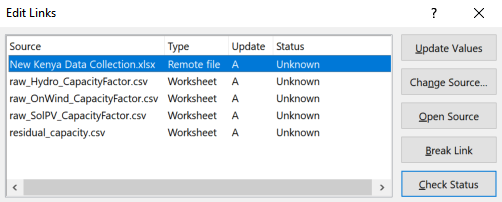  Figure 2: Changing the source of the different spreadsheets linked to the Base Data Collection File according to the user file paths.  Once all the links are working, the user can select the country from the drop-down menu in Tab 1. Model Initiation.  After the relevant data for the country has been selected, the user can break the links to the different worksheets updated earlier (instructions a–b) by clicking on the option of *Break Link*. This should make the spreadsheet work much faster. However, the user must be careful to select the correct values to avoid returning to the spreadsheet to change the country at a later stage, which would need linking the data again by rewriting the formulae- a long and error-prone step for less experienced users. Power Transmission and Distribution output activity ratios Copy the output activity ratio rows for power transmission and distribution from the **TEMBA_Refer.xlsx**ataset from Pappis et al. [13] to the rows in the **3.3 Output Activity Ratios tab of the base SAND file of the selected country**. In this dataset, the codes for power transmission and distribution technologies are ***XXEL00T00X*** and ***XXEL00TDTX,*** respectively, where XX is the country code, which can be obtained from the file **PLEXOS Country Codes.xlsx** from the same repository as the output activity ratio[10]   - In the TEMBA Output Activity Ratios tab, please copy the entire row for XXEL00T00X from column D to column BG and paste it into row 41 of tab 3.3 Output Activity Ratios, starting in column F - In the TEMBA Output Activity Ratios tab, please copy the entire row for XXEL00TDTX from column D to column BG and paste it into row 42 of tab 3.2 Output Activity Ratios, starting in column F  PV, onshore & offshore wind and hydro capacity factors This step relies on linked sources, so filling in some fields is done automatically. The data for the selected country should be inserted in the Raw PV/Onshore Wind/Hydro/Offshore Wind CFs tabs (not the (auto) tabs), and output capacity factors for these technologies should be in the **output capacity factors** tab. The data can be found manually in Cannone et al. [10], in the files “[raw_Hydro_CapacityFactor.csv](https://zenodo.org/record/6142375/files/raw_Hydro_CapacityFactor.csv?download=1)”, “[raw_OffWind_CapacityFactor.csv](https://zenodo.org/record/6142375/files/raw_OffWind_CapacityFactor.csv?download=1)”, “[raw_OnWind_CapacityFactor.csv](https://zenodo.org/record/6142375/files/raw_OnWind_CapacityFactor.csv?download=1)”, “[raw_SolPV_CapacityFactor.csv](https://zenodo.org/record/6142375/files/raw_SolPV_CapacityFactor.csv?download=1)”, “[raw_Wind_CapacityFactor.csv](https://zenodo.org/record/6142375/files/raw_Wind_CapacityFactor.csv?download=1)”. Off-grid capacity Copy and paste the Cumulative/Additions Label for off-grid hydropower and off-grid solar PV for the country’s base SAND file from the **IRENA Installed Capacities data.xlsx** (f[rom Cannone](https://zenodo.org/record/6142375) et al [10]) into the Off-Grid Capacity tab. Note: if the IRENA file has the country but not the relevant off-grid technologies, the values can be assumed to be zero, and no user action is required.   - In the IRENA datasheet, ensure the 'Type' column (column AC) is filtered to '**off-grid.**' - Filter to the selected **country** in the 'IRENA Menu' column (column A) - In the '**Sub-technology**' column, filter to Solar Photovoltaic - Order by Years and then copy the 'Cumulative/Additions Label' column (column AL) into tab **3.7 Off-Grid Capacity**, cells C5–C24 – if there is no data for a year, then put 0, ensure the years match up with the years in column A - Repeat the process but filter out for Renewable Hydropower as the sub-technology, copying the Cumulative/Additions Label column into cells B5–B24 in the tab 3.7 Off-Grid Capacity   Check that the residual capacity rows for PWRSOL002 and PWRHYD004 (rows 920 and 1085) are updated in the **3.7 ResCap data (auto)** tab. If not in the IRENA list, the **TEMBA_Refer.xlsx** file can be used. Go to the residual capacity tab:   - Copy the rows in the residual capacity tab for XXSOV1F01X and XXSOV2F01X from cell B to cell BE. If 0 in all years, no user action is required. - Paste those rows into tab 3.7 ResCap data (auto) tab in rows 1065 and 1066 (PWRSOL002), starting in column I (to the right of the yellow cells). - Ensure that the orange Residual Capacity row for PWRSOL002 (row 1085) is updated if adding values. - Off-grid hydropower is excluded in this case since TEMBA does not include this technology.  On-grid residual capacity This step relies on linked sources, so filling in some fields is done automatically. Check that on-grid residual capacity data for the selected country is in the 3.7 ResCap data (auto) tab. Focus on checking between rows 724–1197 as this is where the power plant technologies are (all begin with PWR).  Find the raw on-grid residual capacity data for the selected country in the file **residual capacity.csv** from Brinkerink and Deane [10][14] and check that there are no more than 11 power plants of each type for the selected country, as this is the maximum number permitted by the spreadsheet. If there are more than 11 rows, extra rows can be manually added, and extra data copied directly from the CSV file.  A few manual adjustments are needed:   - For hydropower (rows 852–920), move any plants in the PWRHYD001 group to PWRHYD002 if they have a capacity between 0.01–0.1 GW and to PWRHYD003 if less than 0.01 GW (capacity is in column H). To move a plant, copy the entire row for that plant from column E to column AR, and paste it in the corresponding cells of the intended technology group. - For oil power plants (rows 985–1016), all residual capacity is automatically inserted as PWROHC001 (LFO plant). However, to find more information, it can be done online research of the Power Plant's name, and any plants that do not belong to the right category should be moved to PWROHC002 (HFO gas turbine) using the method above. If no information can be found, leave the plant where it is. - The same should be done for gas power plants (rows 921–952), moving plants from PWRNGS001 (CCGT) to PWRNGS002 (OCGT) if it can be found out that the plant is an OCGT. If no reliable data is found, make no changes. Useful sites for checking the type of power plant are [15] [16]; these can be searched by power plant name or by country and cover both oil and gas.  Refinery and Transmission & Distribution residual capacity Copy and paste the residual capacity for the following technologies from the **residual capacity tab** of the TEMBA_Refer.xlsx dataset from Pappis et al. [17] into the **3.7 ResCap data (auto)** tab below. If values of 0 are seen for all years, no user action is required, as no residual capacity data are available for that specific power plant type. If values are non-zero, copy the entire row in TEMBA from column B to BE:   - The row for XXCRUDRE1X in TEMBA (where XX is country code) should be copied to the Residual Capacity row for UPSREF001 in row 3181 in tab 3.7 ResCap data (auto), starting in column I - The row for XXCRUDRE2X in TEMBA (where XX is country code) should be copied to the Residual Capacity row for UPSREF002 in row 3197 in tab 3.7 ResCap data (auto), starting in column I - The row for XXEL00T00X in TEMBA (where XX is country code) should be copied to the Residual Capacity row for PWRTRN in row 1117 in tab 3.7 ResCap data (auto), starting in column I - The row for XXEL00TDTX in TEMBA (where XX is country code) should be copied to the Residual Capacity row for PWRDIST in row 835 in tab 3.7 ResCap data (auto), starting in column I - Note, the rows that are pasted into should be the ones shaded in orange.   Note the PWR technologies (rows 724–1197 in tab 3.7 ResCap data (auto)) that do not have any residual capacity in the country, as this is needed for Step 7. Do not include the following technologies: PWRTRN, PWRDIST, PWRTRNIMP, PWRTRNEXP. Repeat these steps for the refinery technologies: UPSREF001 and UPSREF002 (rows 3166 to end) (XXCRUDRE1X & XXCRUDRE2X in TEMBA, respectively). Capacity Constraints In the 3.8 Capacity & Inv Constraints tab, set the **Total Annual Max Capacity Investment** for 2015–2020 to 0 (column E to column J) for power generation and refinery technologies that have 0 residual capacity in the country, using the list made in Step 7. Do not do this for the following technologies: PWRTRN (row 439), PWRDIST (row 440), PWRTRNIMP (row 437), PWRTRNEXP (row 443).  If the country has no offshore wind potential, put 0 for the **Total Annual Max Capacity** for offshore wind all years (PWRWND002, row 37, 0 should be input from column E to BH). Highlight the rows where these constraints have been added in green, as they will be pasted to SAND later. Demands This step relies on linked sources, so filling in some fields is done automatically, but only partially. Check rows 7–11 in the 4. TEMBA Demands Data tab have been filled in with data (rows 7–11, columns C–BF). Make sure this process is done in the **4. TEMBA Demands Data tab** (do **not** use the the 4. TEMBA Demands Data **(auto)** tab).  For IEA countries, insert the final consumption (in PJ; convert from TJ if needed) for each fuel in each sector in the country for 2015–2018 into the table (rows 15–30, columns B to E) in the 4. TEMBA Demands Data tab from the IEA Sankey Diagram [18], marking 0 if there is no consumption for that fuel/sector. Key points:   - For this step, ensure the 'Final Consumption' option is selected rather than 'Balance' in the navigation pane on the left of the IEA website - Ensure the unit is changed to PJ/TJ at the top of the diagram, and be sure to convert to PJ if it is in TJ (divide by 1,000) - The consumption in each sector can be seen by clicking on the sector on the diagram, which opens a pie chart - Change the year by dragging the slide along the bottom   For non-IEA countries:   - Find the United Nations Energy Balance for the selected country on the UN website [19] (PDFs for groups of countries in alphabetical order). - For these countries, insert data in the 2018 and 2017 columns of the table in the 4. TEMBA demands data tab (columns D and E, rows 15–30). The UN energy balances are in TJ; so divide by 1,000 to convert to PJ when inserting data. The top section of the UN energy balance is usually marked as 2018, then 2017 data are below – but check this for the selected country. - Look at the data in the 'Final energy consumption' sections of the energy balance for 2017 and 2018. For industry, use the values for 'Manufacturing, const, mining'. For transport use the values for 'Transport'. For commerce use the values 'Commerce and public services’. For residential use the values for 'Households'. Use 'All Oil' for oil products; sum the values for 'Primary biofuels/Waste' and 'Charcoal' for biofuels and waste. - Leave the columns for 2015 and 2016 (columns B and C) blank, so they will not be considered in the average calculated in column F.   Demands will then be automatically calculated: check in the 4.1 Accumulated Annual Demand tab that the rows for TRAMCY, TRACAR, TRABUS, INDHEH, INDHEL, RESCKN, COMHEL, and RESHEL have been filled in (shaded in green, between rows 19–29), and in the 4.2 Specified Annual Demand tab that the rows for INDELC, RESELC, and COMELC (rows 22, 25, & 27) have been filled in (shaded in green). These demands consider the input activity ratios of the technologies used to deliver them; for example, the demand for motorcycles is initially calculated in terms of oil demand based on the input data, and this is then converted into the final energy demand for motorcycles considering the efficiency of the oil motorcycle technology. Electricity demand profile Copy and paste the hourly electricity demand profile for the selected country from the PLEXOS All Demand UTC 2015.tab dataset downloadable from Brinkerink and Deane [14] into the 4.2 Elc Demand Profile Raw Data tab.   - In PLEXOS the countries are along the tab (countries organized in columns), with the region code (AF for Africa), followed by the country code), copy the whole column starting from row 2 to row 8761. - Paste the column into tab **4.2 Elc Demand Profile Raw Data** starting in cell B4 (marked in yellow).   Go to the **4.2 Specified Dem Profile Calc** tab and to the rows for RESELC, COMELC, and INDELC (rows 21, 24, and 26). Adjust the value in column L until the value in column M is exactly equal to 1. Only small adjustments are needed: e.g., if the value in column M is 1.007, first try adjusting the value in column L to 0.98, then make further small adjustments if needed. Check that Specified Demand Profiles have been calculated for RESELC, COMELEC, and INDELC in the 4.2 Specified Demand Profile Output tab (columns W, Z, AB). Import & Export activity limits For IEA countries: Insert the amounts of imported and exported electricity (PJ) from the IEA Sankey diagram [18] for the country for 2015–2018 into the TotalTechnologyAnnualActivityUpperLimit rows for PWRTRNIMP (row 238) and PWRTRNEXP (row 244) in tab 5.1 Activity in columns F (2015) to I (2018). Columns beyond column I are automatically calculated based on the values entered in columns F to I. Important points:   - For this Step ensure the **'Energy Balance'** option is selected in the left-hand navigation pane on the IEA Sankey website. - Ensure that the unit is set to PJ/TJ as in Step 8, and carry out unit conversions if needed. - Data can also be obtained from the IEA's energy balance tables [18]. - If there is no data, set to 0.   For non-IEA countries: open the UN energy balance for the selected country used in the earlier demands step. UN data are in TJ, which must be divided by 1,000 to convert to PJ. Go to tab 5.1 Activity. Insert the amounts of imported and exported electricity from the energy balance in 2017 and 2018 into the TotalTechnologyAnnualActivityUpperLimit rows for PWRTRNIMP (row 238) and PWRTRNEXP (row 244) in column H for 2017 and column I for 2018. Electricity imports and exports are found in the UN energy balance in the top section for each year in the rows for 'Imports' and 'Exports' under 'Electricity'. Columns beyond column I are automatically calculated based on the values entered in columns F to I. Important points:   - The values for 2017 must be inserted into the columns for 2015 and 2016 (columns F and G) – it is assumed that imports & exports remain similar across years. - Do not include the minus sign found before the values for exports in the UN energy balance data. - Remember that the UN data are in TJ and must be divided by 1,000 to convert to PJ.  Renewable and fossil resources Insert the estimated renewable energy potentials in the selected country into the Data in Brief Tables 8 & 9 Tab from the sources indicated in Table 2 below. Some notes:   - If the table does not contain a potential for geothermal, insert a value of 0 for the geothermal potential (geothermal potentials are only included in the report for Eastern/Southern Africa and can be assumed to be 0 elsewhere). - If there is a dash in the report, assume 0. - When using the World Small Hydropower Development Report tables, select the value from the far-right column titled "Potential (<10MW)" or "Potential Capacity" for Middle Africa. - When using the **IRENA Southern African Power Pool (SAPP) and (West African Power Pool) WAPP** reports for hydropower potential, the potential is sourced from the column titled 'Identified Projects (MW)'. Calculate the sum of the last number with the Existing Capacity (MW) value, as the aim is to capture the overall potential. Ignore the small hydropower potentials in these reports, as these are sourced from the report above. When using the **IRENA Eastern & Southern Africa report**, hydropower potential is taken from the column titled 'Hydropower (MW), Potential'.   Table 2: Data sources for Renewable and fossil fuel resources for African countries   \| Country \| Small Hydro \| Hydro & Geothermal (where applicable) \| PV, CSP, Wind \| \| --- \| --- \| --- \| --- \| \| Angola, Botswana, DRC, Eswatini/Swaziland, Lesotho, Malawi, Mozambique Namibia, South Africa, Zambia, Zimbabwe \| World Small Hydropower Development Report (Pages 9, 83, 119, 146, 175) [20] \| IRENA Southern African Power Pool (SAPP) report (Table 2, page 21) [21] \| Hermann et al. 2014 (Table 10 pages 35–36) [22] \| \| Benin, Burkina Faso, Cote D'Ivoire, Gambia, Ghana, Guinea, Liberia, Mali, Niger, Nigeria, Senegal, Sierra Leone, Togo \| World Small Hydropower Development Report (Pages 9, 83, 119, 146, 175) [20] \| IRENA (West African Power Pool) WAPP report (Table 4, page 31) [23] \| Hermann et al. 2014 (Table 10 pages 35–36) [22] \| \| Burundi, Ethiopia, Kenya, Rwanda, Sudan, Uganda, Tanzania, Djibouti, Egypt, Libya \| World Small Hydropower Development Report (Pages 9, 83, 119, 146, 175) [20] \| IRENA Eastern & Southern Africa report (Table 10 on page 35) [24] \| Hermann et al. 2014 (Table 10 pages 35–36) [22] \| \| Cameroon, Central African Republic, Chad, Republic of Congo, Equatorial Guinea, Eritrea, Gabon, Mauritania, Somalia, Guinea-Bissau \| World Small Hydropower Development Report (Pages 9, 83, 119, 146, 175) [20]: note Chad & Eritrea are absent, assume 0. \| TEMBA report (Table 12 on page 89) [25] \| Hermann et al. 2014 (Table 10 pages 35–36) [22] \|   Insert the estimated fossil fuel reserves in the country into the table in the tab Data in Brief Tables 8 & 9 from Table 11 on page 88 of the TEMBA report [25]. If there is a dash, assume 0. If the country is not in the table, it is assumed there are no domestic reserves. In this case, insert 0 for coal, gas, and oil.  Check that total technology model period activity upper limits have been added for MINOIL, MINNGS, and MINCOA in the 5.1 Activity tab (row 601, 605, 611), and that total annual max capacity limits have been updated for PWRGEO (row 24) and PWRHYD001-004 (rows 33–35) in the tab 3.8 Capacity & Inv Constraints if applicable. |
| --- | --- | --- | --- | --- | --- | --- | --- | --- | --- | --- | --- | --- | --- | --- | --- | --- | --- | --- | --- | --- |

Table 3: How to transfer Region-Specific data to SAND Interface

| **Data** | **Location of Data in Data Collection File** | **How to add/update data in SAND Interface for the following Parameter** |
| --- | --- | --- |
| Discount Rate (Default Value) | 1. Model initiation | Discount Rate |
| Depreciation Methods (Default Value) | 1. Model initiation | Depreciation Methods |
| Year Split Values (Default Values) | 2. Year Split | Year Split |
| Technologies’ Costs | 3.1 Technology Costs, rows 3–80 (capital costs), rows 202–279 (fixed costs), rows 401–478 (variable costs) | CapitalCost, FixedCost and VariableCost for all the technologies |
| Input Activity Ratios | 3.2 Input Activity Ratios, rows 3–80 (excluding N.A.) | InputActivityRatio for all the technologies where the value is not N.A. |
| Output Activity Ratios | 3.3. Output Activity Ratios, rows 3–80 | OutputActivityRatio for all the technologies listed |
| Capacity To Activity Unit | 3.4 Capacity to Activity Unit, rows 3–80 | CapacityToActivityUnit for all the technologies listed |
| Operational Life | 3.5. Operational Life, rows 3–80 | OperationalLife for all the technologies listed |
| Emissions Activity Ratio | 6.1. Emissions Activity Ratio, rows 4–15 | EmissionsActivityRatio for all the technologies where the value is different from 0. |
| Capacity Factors for power plants with non-variable production | 3.6 Output Capacity Factors, column S, U, W, X, Y, Z, AA, AD, AE, AK, and AP | CapacityFactor for each power plant with a non-variable production (PWRBIO001, PWRCOA001, PWRGEO, PWROHC001, PWROHC002, PWRNGS001, PWRNGS002, PWRCPS001, PWRCSP002, PWRNUC, PWROHC003). Leave the default value of 1 for all the other technologies. |

Table 4: How to transfer country-specific data to SAND Interface

| **Data** | **Location of Data in Data Collection File** | **Technologies/Fuels** |
| --- | --- | --- |
| Output Activity Ratios for Transmission and Distribution Technologies | 3.3 Output Activity Ratios, rows 41 and 42, columns F to BI | PWRTRN, PWRDIST – in SAND, make sure to add the data for PWRTRN on the row marked ELC002 and PWRDIST on the row marked ELC003. |
| Capacity Factors for power plants with variable energy production | 3.6 Output Capacity Factors, between columns AC to BX, row 3 to row 98. *These should be extended to 2070 in SAND* | PWRSOL001, PWRHYD001, PWRHYD002, PWRHYD003, PWRWND001, PWRWND002 (only if the country has offshore wind potential)  PWRHYD004, PWRSOL001S, PWRWND001S |
| Residual Capacity for Transmission, Distribution, and Refineries Technologies | 3.7 ResCap data (auto), between rows 724–1197 for PWR techs and rows 3181 for UPSREF001 and row 3197 for UPSREF002, columns I to AR. (see note above on adding residual capacity too). | PWRTRN, PWRDIST, and all PWR technologies that have residual capacity in the country, as well as UPSREF001/UPSREF002 if they have residual capacity in the country. |
| Total Annual Max Capacity for Hydropower, Geothermal, and Offshore wind power plants | 3.8 Capacity & Inv Constraints, rows 33, 34, 35, (37 for offshore wind), 75, and 24, columns E to BH. | PWRHYD001, PWRHYD003, PWRHYD003, PWRHYD004, PWRGEO. PWRWND002 if it has been set to 0 for the country as offshore wind is not possible. |
| Total Annual Max Capacity Investment | 3.8 Capacity & Inv Constraints, between rows 418–473 depending on the country, columns E to BH. | For 2015–2020 for any of the PWR or UPS technologies that have been set to 0 for because there was no residual capacity (these cells should be highlighted in green when prepping the data) |
| Accumulated Annual Demand | 4.1 Accumulated Annual Demand. Rows 19, 20, 21, 23, 24, 26, 28, and 29, columns D to BG | Fuels: TRAMCY, TRACAR, TRABUS, INDHEH, INDHEL, RESCKN, COMHEL, RESHEL |
| Specified Annual Demand | 4.2 Specified Annual Demand. Rows 22, 25, and 27, columns D to BG | INDELC, RESELC, COMELC |
| Specified Annual Demand Profile | 4.2 Specified Dem Profile Output. Columns W, Z and AB, rows 4 to 99. *extend these values to 2070 in SAND* | INDELC, RESELC, COMELC |
| Total Technology Annual Activity Upper Limit | 5.1 Activity, rows 238 and 244, columns F to BI. | PWRTRNIMP, PWRTRNEXP |
| Total Technology Annual Activity Lower Limit | 5.1 Activity, rows 39 and 45, columns F to BI. | PWRTRNIMP, PWRTRNEXP |
| Total Technology Model Period Activity Upper Limit | 5.1 Activity, rows 601, 605, 611, column E. | MINOIL, MINNGS, MINCOA |

**Box 2** explains how to transfer the data from the Data Manipulation and Collection Tool to the clicSAND Excel Interface in an automated way with a Python code. The methodology to create modelling scenarios in a manual and automatic way is explained in **Box 3** and **Box 4** respectively.

| **Box 2. Automated Transfer with Python code**   1. **1. Copy the following relevant files to a local directory**.  - The data preparation Python script ccg_data_to_sand.py, which includes a list of Python packages requirements [8]. - The "Base Data Collection File.xlsx" [1]. - The 'Starter Kit - List of Countries and Regions.xlsx' file from [9].  1. **2. Prepare a virtual Python environment.**  - Open the terminal. - Navigate to the relevant directory.   cd /path/to/ccg_data_to_sand_directory   - Create a virtual environment (recommended).   python3 –m venv venv  source venv/bin/activate  Note: example command lines are given using python3, though users can also complete the same steps using python.   - Download the package dependencies.   python3 -m pip install -r requirements.txt  For further help (or if pip extension is not already installed), please check: https://packaging.python.org/tutorials/installing-packages/   1. **3. Update user inputs.** 2. In the ccg_data_to_sand.py Python file, the user should update:    - path_input: the path to the folder containing the input data files “Data Collection File.xlsx' and 'Starter Kit - List of Countries.xlsx'.    - path_SAND_out: the path to the folder where the "{country_name} Base SAND parameters.csv" file will be saved (default same as path_input).    - countries: list of countries for which SAND files should be created. Note this should be written in CAPITAL letters. 3. **4. Run Python script.** 4. Run the script twice, once to obtain the link of the adequate base SAND file to download as will be printed on the screen, and a second time to perform the data preparation step.  - Open the terminal.   - Activate the relevant virtual environment (if applicable)   cd /path/to/ccg_data_to_sand.py  source venv/bin/activate   - - Run the script.   python3 ccg_data_to_sand.py   1. **5. Copy values into Parameters Sheet.**    - Create a new empty base SAND Excel file by copying ‘Base SAND.xlsm’ [26] and renaming it to “New_{country_name}_Base_SAND.xlsm".    - Ensure all rows are visible and no filtering is active in country Base SAND.xlsm. Macros for the file may need to be enabled.    - Open the “{country_name} Base SAND parameters.csv" file that was generated by the Python script.    - Copy the contents from the entire CSV file.    - Paste values only into the Parameters tab of the “New_{country_name} _Base_SAND.xlsm” file. Note: This might only work in the desktop Excel version, not the web app. |
| --- |

| **Box 3. Manual Scenarios Creation**  **1. Fossil Future**   - Create a copy of the 'Scenario Constraints' file [27] and name it 'Your_Country_Name_Scenario_Constraints'. - Follow the instructions in the ‘Input Data’ tab of the ‘Your_Country_Name_Scenario_Constraints’ file to add a few bits of data for the country from the Data Collection and Manipulation File. - The Data Collection and Manipulation file can now be closed. - Go to the Fossil Future tab in the Country Scenario Constraints file and follow the instructions to set up the Fossil Future scenario in a copy of the Base SAND file named “Your-Country_Name_FF_SAND (all instructions needed should be in this tab). Then download the Country FF SAND file to a local computer – it is suggested to create a folder called ‘Runs’ on the Desktop and put this (and everything else from now on) in here.   **2. Least Cost Scenario**   - Go back to the “Country Scenario Constraints” file and follow the instructions to set up the Least Cost scenario in the Least Cost tab. - Once set up, download the “Country LCv1 SAND” file and run it for the FF scenario. Repeat the process above to set up the results database, and results excel file for LCv1 – creating a copy of the results database and “Results Template”, downloadable from here [26], and naming them “Country LCv1 Results Database/Results”. Remember to change the file name of the results file produced by clicSAND to remove the spaces before importing to the database and select all the relevant technologies for each graph in the Results Excel file. - Then go back to the “Country Scenario Constraints” file and follow the instructions to set up “Country LCv2” before running it and creating the results files in the same way.   **3. Net Zero Scenario**   - Go back to the “Country Scenario Constraints” file and follow the instructions to set up the Net Zero scenario in the Net Zero tab. - Once set up, download the “Country NZv1 SAND” file and run it has done for the FF and LC scenarios. Repeat the process above to set up the results database, and results excel file for NZv1. - Then go back to the "Country Scenario Constraints” file and follow the instructions to set up “Country NZv2” before running it and creating the results files in the same way.   - CCG_SAND_Scenario_NZv2.py |
| --- |

| **Box 4. Automated Scenario Creation with Python Code**  **1.** Copy the following data preparation scripts (from Cannone et al. [11] on Zenodo) to a local directory:   - - CCG_SAND_Scenario_FF.py   - CCG_SAND_Scenario_LCv1.py   - CCG_SAND_Scenario_LCv2.py   - CCG_SAND_Scenario_NZv1.py   - CCG_SAND_Scenario_NZv2.py   **2.** Create a copy of the 'Scenario Constraints' file [27] and name it 'Your_Country_Name_Scenario_Constraints'. Save it to a local directory.  **3.** Update user inputs: in the CCG_SAND_Scenario_FF.py Python file, the user should update:   - - path_countries: path to the base folder that contains other folders. Need not be used if the folder structure is different.   - path_SAND_out: path to the folder with SAND CSV files   - path_scenarios: path to the folder with Scenario file   - countries: list of countries for which SAND CSV files should be created   **4.** Run Python scripts.   - - Open the terminal.   - Activate the appropriate virtual environment created in the previous section to create the model (if applicable). This environment should already have the necessary packages installed.   cd /path/to/ccg_data_to_sand.py  source venv/bin/activate   - - Type python3 cd /path/to/ CCG_SAND_Scenario_FF.py, then press ENTER.   **5.** Copy values into the Parameters sheet.   - - Create a new empty FF SAND.xlsm file by copying the country_base_SAND.xlsm and renaming country_FF_base_SAND.xlsm.   - Open the new country_FF_SAND.xlsm file in Excel and ensure all rows are visible and no filtering is active.   - Open the County FF SAND parameters.csv file in Excel.   - Copy the contents of the entire CSV file.   - Paste using the “values” option into the Parameters tab of “Country_FF_SAND.xlsm” file. Note: This might only work in the desktop Excel version, not the web app.   **6.** Repeat Steps 3–5 for the Least Cost version 1 and 2 scenarios and Net Zero version 1 and 2 scenarios using the following python files instead of the CCG_SAND_Scenario_FF.py file:   - - CCG_SAND_Scenario_LCv1.py   - CCG_SAND_Scenario_LCv2.py   - CCG_SAND_Scenario_NZv1.py |
| --- |

**References**

[1] C. Cannone *et al.*, “CCG Starter Kits - Base SAND file for Africa,” *CCG Starter Kits Supporting Data and Scripts*, 2022. .

[2] C. Cannone *et al.*, “Starter Kits - Base SAND file for South America,” *CCG Starter Kits Supporting Data and Scripts*, 2022. .

[3] C. Cannone *et al.*, “Starter Kits - Base SAND file for Asia,” *CCG Starter Kits Supporting Data and Scripts*, 2022. .

[4] C. Cannone *et al.*, “CCG Starter Kits - Base SAND file for Africa Coal and Natural Gas Scenario,” *CCG Starter Kits Supporting Data and Scripts*, 2022. .

[5] C. Cannone *et al.*, “CCG Starter Kits - Base SAND file for Africa Natural Gas Scenario,” *CCG Starter Kits Supporting Data and Scripts*, 2022. .

[6] C. Cannone *et al.*, “CCG Starter Kits - Base SAND file for Asia - Coal and Natural Gas Scenario,” *CCG Starter Kits Supporting Data and Scripts*, 2022. .

[7] C. Cannone *et al.*, “CCG Starter Kits - Base SAND file for South America- Coal and Natural Gas Scenario,” *CCG Starter Kits Supporting Data and Scripts*, 2022. .

[8] F. Charbonnier *et al.*, “CCG Starter Kits - Script for csv creation with base SAND data for Starter Kits,” *CCG Starter Kits Supporting Data and Scripts*, 2022. .

[9] C. Cannone *et al.*, “Starter Kit - List of Countries and Regions,” *CCG Starter Kits Supporting Data and Scripts*, 2022. .

[10] “CCG Starter Kits - Technology-specific data for Base SAND file | Zenodo.” https://zenodo.org/record/6142375#.Y-4ukXbMJPY (accessed Feb. 16, 2023).

[11] C. Halloran *et al.*, “Starter Kits - Scripts for SAND scenarios,” *CCG Starter Kits Supporting Data and Scripts*, 2022. .

[12] “Starter Kits – Climate Compatible Growth.” https://climatecompatiblegrowth.com/starter-kits/ (accessed Aug. 24, 2021).

[13] I. Pappis, V. Sridharan, W. Usher, and M. Howells, “KTH-dESA/jrc_temba: TEMBA 2.1,” Jun. 2021, doi: 10.5281/ZENODO.4889373.

[14] M. Brinkerink and P. Deane, “PLEXOS-World 2015,” 2020.

[15] “Global Energy Monitor.” https://www.gem.wiki/Main_Page (accessed Feb. 21, 2022).

[16] “List of Gas PowerPlants - GEO.” http://globalenergyobservatory.org/list.php?db=PowerPlants&type=Gas (accessed Feb. 21, 2022).

[17] I. Pappis, V. Sridharan, W. Usher, and M. Howells, “KTH-dESA/jrc_temba: TEMBA 2.0 (Version v2.0.3) [Data set],” 2021. doi: http://doi.org/10.5281/zenodo.4633042.

[18] International Energy Agency, “IEA Sankey Diagram,” 2019. .

[19] “UNSD — Energy Statistics.” https://unstats.un.org/unsd/energystats/pubs/balance/ (accessed Jun. 03, 2020).

[20] United Nations, “World Small Hydropower Development Report 2019,” 2019.

[21] IRENA, “Southern African Power Pool: Planning and Prospects for Renewable Energy,” 2013. https://www.irena.org/documentdownloads/publications/sapp.pdf (accessed Oct. 23, 2020).

[22] S. Hermann, A. Miketa, and N. Fichaux, “Estimating the Renewable Energy Potential in Africa,” Abu Dhabi, 2014.

[23] I. Renewable Energy Agency, “Planning and prospects for renewable power: WEST AFRICA 2018,” 2018, Accessed: Feb. 21, 2022. [Online]. Available: www.irena.org.

[24] IRENA, “Analysis of Infrastructure for Renewable Power in Eastern and Southern Africa,” Abu Dhabi, 2015.

[25] I. Pappis *et al.*, “Energy projections for African countries service contract 936531.”

[26] “Blank SAND Interface for clicSAND Software | Zenodo.” https://zenodo.org/record/6203284#.YhOM4OjMJPY (accessed Feb. 21, 2022).

[27] “Scenario Constraints Excel Template for Starter Data Kits | Zenodo.” https://zenodo.org/record/6204654#.YhOcv-jMJPY (accessed Feb. 21, 2022).
